# Intermetallic Cu_5_Zr Clusters Anchored on Hierarchical Nanoporous Copper as Efficient Catalysts for Hydrogen Evolution Reaction

**DOI:** 10.34133/2020/2987234

**Published:** 2020-02-20

**Authors:** Hang Shi, Yi-Tong Zhou, Rui-Qi Yao, Wu-Bin Wan, Qing-Hua Zhang, Lin Gu, Zi Wen, Xing-You Lang, Qing Jiang

**Affiliations:** ^1^Key Laboratory of Automobile Materials (Jilin University), Ministry of Education, and School of Materials Science and Engineering, Jilin University, Changchun 130022, China; ^2^Beijing National Laboratory for Condensed Matter Physics, The Institute of Physics, Chinese Academy of Sciences, Beijing 100190, China

## Abstract

Designing highly active and robust platinum-free electrocatalysts for hydrogen evolution reaction is vital for large-scale and efficient production of hydrogen through electrochemical water splitting. Here, we report nonprecious intermetallic Cu_5_Zr clusters that are in situ anchored on hierarchical nanoporous copper (NP Cu/Cu_5_Zr) for efficient hydrogen evolution in alkaline medium. By virtue of hydroxygenated zirconium atoms activating their nearby Cu-Cu bridge sites with appropriate hydrogen-binding energy, the Cu_5_Zr clusters have a high electrocatalytic activity toward the hydrogen evolution reaction. Associated with unique architecture featured with steady and bicontinuous nanoporous copper skeleton that facilitates electron transfer and electrolyte accessibility, the self-supported monolithic NP Cu/Cu_5_Zr electrodes boost violent hydrogen gas release, realizing ultrahigh current density of 500 mA cm^−2^ at a low potential of -280 mV versus reversible hydrogen electrode, with exceptional stability in 1 M KOH solution. The electrochemical properties outperform those of state-of-the-art nonprecious metal electrocatalysts and make them promising candidates as electrodes in water splitting devices.

## 1. Introduction

Global climate change and environmental pollution caused by excessive CO_2_ emission have raised tremendous demands for new energy sources with renewability and sustainability [1–3]. Hydrogen, as a high-density energy carrier in clean and sustainable energy framework of water-hydrogen interconversion, has long been considered as one of the most promising alternatives to traditional fossil fuels for meeting future energy needs [4, 5]. Electrochemical water splitting driven by renewable electricity from plentiful solar, wind, and hydro resources is an attractive and feasible approach to large-scale production of hydrogen [4, 6, 7] showing genuine potential in replacing commercial technologies (such as catalytic steam methane reforming, partial oxidation, and coal gasification) that are neither environmentally friendly nor cost-effective because of vast CO_2_ emission and harsh high-temperature requirements. During the process of water electrolysis at room temperature, the hydrogen evolution reaction (HER) is a crucial step but suffers from a sluggish kinetics [6, 8], particularly in alkaline electrolytes [7, 9–14], which urgently calls for robust and efficient catalytic materials to minimize the HER overpotential at high current densities [6–16]. Highly conductive precious metals, including platinum (Pt) and ruthenium (Ru), are at present the best HER catalysts by virtue of their appropriate hydrogen-binding energy (HBE) values that are conducive to the adsorption of reactive hydrogen intermediates (H^∗^) and their recombination into hydrogen molecules [6, 17, 18]. However, their scarcity, expensive cost, and poor durability essentially impede the practical use of Pt and Ru for scalable hydrogen production even in various nanostructured alloys or composites with high specific surface areas [19–23]. To address these issues, it is crucial to explore Earth-abundant nonprecious metal electrocatalysts with superior HER catalysis towards a successful hydrogen economy [7, 13, 15, 16, 24]. In view that monometallic transition metals (e.g., Fe, Co, Ni, Mo, and W) generally have too weak or too strong HBE values [6, 17, 25], considerable research efforts have been focused on developing their compounds (such as carbides [26, 27], nitrides [28, 29], phosphides [30, 31], sulfides [32, 33], oxides [34], or selenides [35]). Initial strides have been made in improving their HER catalysis by nanostructuring to increase the number of active sites or/and making use of C, N, P, S, or Se heteroatoms to enhance intrinsic activity via adjusting the HBE values to balance the H^∗^ adsorption/desorption [6, 13, 15, 16]. Unfortunately, few of these materials operate at low overpotentials due to either insufficient catalytic activity or poor electron transfer caused by electron localization of electrovalent bonds. Even though nanostructuring is expected to ameliorate electron transferability by shortening the distance from electroactive to conductive materials, immobilizing these low-dimensional nanostructures on planar current collectors to construct macroscale electrodes inevitably leads to durability and efficiency issues [33, 35–37], unsatisfying practical requirements of large-scale hydrogen production, i.e., low working overpotential and long-term stability at high current density [26, 29, 30, 38–40]. Therefore, it is desirable to design and develop novel low-cost electrocatalytic materials with a rational electrode architecture that not only withstands violent gas evolution but has abundant high active sites and facilitates fast electron transfer and electrolyte transport to minimize the overpotential.

In this study, we report self-supported monolithic electrocatalytic materials, which are composed of high-density intermetallic Cu_5_Zr clusters in situ integrated on bicontinuous nanoporous Cu current collector (NP Cu/Cu_5_Zr), for highly efficient production of hydrogen in alkaline media. Therein, the constituent Cu_5_Zr clusters serve as active sites to boost the sluggish reaction kinetics of the HER as a result of hydroxylation of surface Zr atoms activating their nearby Cu-Cu bridge sites with an appropriate HBE conducive to the H^∗^ adsorption/desorption. The specific current density of Cu_5_Zr clusters reaches 3.1 mA cm^−2^, ~300-fold higher than that of bare Cu, at the potential of -200 mV versus a reversible hydrogen electrode (RHE). Because bicontinuous nanoporous Cu skeleton is conductive and steady enough to facilitate fast electron transfer and withstand violent hydrogen gas evolution, the NP Cu/Cu_5_Zr electrodes exhibit superior HER catalysis in 1 M KOH solution, with a low onset overpotential of ~30 mV, a small Tafel slope of ~68 mV dec^−1^ and a long-term durability at high current densities. Furthermore, it takes the NP Cu/Cu_5_Zr only a low potential -280 mV versus RHE to achieve a current density of as high as 500 mA cm^−2^, outperforming the state-of-the-art nonprecious metal-based catalysts. These impressive electrochemical properties make them promising alternatives to precious metal-based catalysts for efficient water electrolysis in basic solutions.

## 2. Results and Discussion

### 2.1. DFT Theoretical Calculations

**Figure 1:**
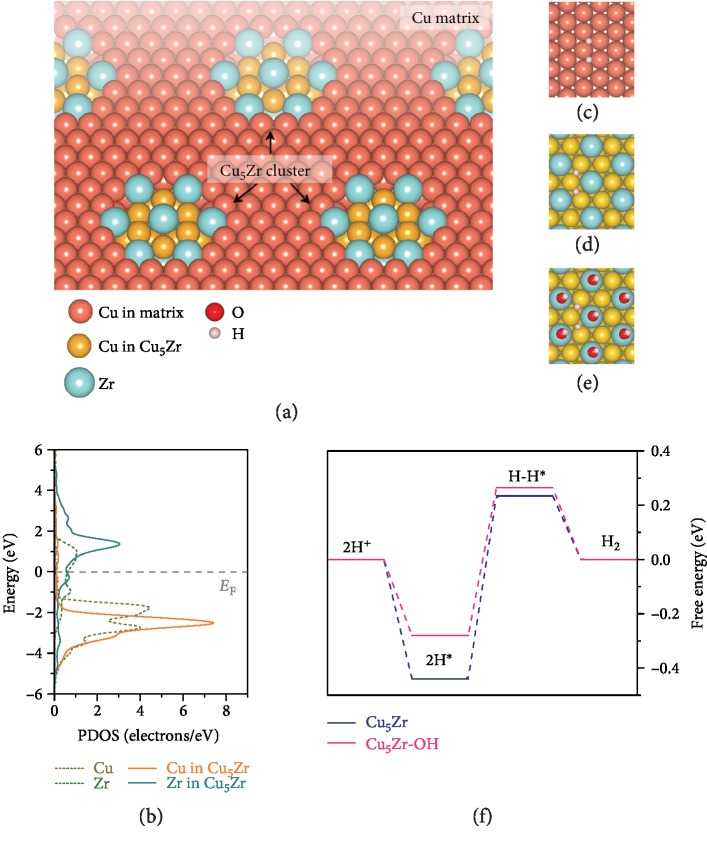
DFT calculation of Cu_5_Zr intermetallic compound. (a) Schematic illustrating Cu matrix embedded with Cu_5_Zr clusters. Cu atoms in the matrix and Cu_5_Zr clusters in orange and golden, Zr atoms in cyan, O in red, and H in magenta, respectively. (b) Partial density of states (PDOS) of Cu atom in Cu(111), Zr atom in Zr(111), and Cu and Zr atoms in Cu_5_Zr(111). (c–e) Optimized atomic structures of facets of Cu(111) (c), Cu_5_Zr(111) (d), and Cu_5_Zr-OH(111) (e) with hydrogen adsorption (H^∗^). (f) Free-energy diagram of the Tafel route for the HER on Cu_5_Zr and Cu_5_Zr-OH surfaces.

Cu is a classic metal with high conductivity but usually exhibits poor catalytic activity. Here, we devise a highly efficient HER copper catalyst, which is modified by Zr via the formation of Cu-Zr intermetallic clusters (Figure 1(a)). Owing to the large electronegative difference between Cu and Zr, there forms structurally ordered Cu_5_Zr intermetallic compound [41–44], one of the most thermodynamically stable phases with a face-centered cubic (fcc) structure in Cu-Zr system, in the Cu-rich component region [41–44]. On the basis of Cu_5_Zr(111), first-principles density functional theory (DFT) calculations demonstrate that Zr atoms can effectively activate their surrounding negative-valence Cu atoms. As shown in partial density of states (PDOS) of Cu_5_Zr(111) (Figure 1(b)), the center of *d* bands of the Cu_5_Zr is shifted up toward the Fermi energy relative to bare Cu(111). Although surface Cu atoms tend to be oxidized into Cu(OH)_2_ layer in the ambient surroundings, they are reduced to the metallic state in the HER potential range because of the Cu^2+^/Cu equilibrium potential of 0.61 V at pH = 14 [45]. This is demonstrated by the DFT simulation on the Cu_5_Zr(111), on which except for the Zr atoms, the Cu atoms do not adsorb hydroxyl group (HO^∗^) at 0 V vs. RHE (supplementary [Supplementary-material supplementary-material-1]). Distinguished from the hollow-site adsorption of Cu(111) (Figure 1(c)), the incorporation of Zr enables the Cu-Cu bridge sites of the Cu_5_Zr(111) surface to adsorb hydrogen atoms, with a calculated HBE per H atom of -0.44 eV (Figure 1(d), supplementary [Supplementary-material supplementary-material-1] and [Supplementary-material supplementary-material-1]). While it is thermodynamically favorable to adsorb HO^∗^ on the surface Zr atoms (Cu_5_Zr-OH) in the HER potential range (Figure 1(e) and supplementary [Supplementary-material supplementary-material-1]), the hydroxylation does not adjust the optimal adsorption sites except for slightly weakening the HBE value to -0.36 eV (supplementary [Supplementary-material supplementary-material-1] and [Supplementary-material supplementary-material-1]). Such unique configuration is more favorable for the Cu_5_Zr-OH(111) to accelerate the HER kinetics than the clean Cu_5_Zr(111), suggesting a superior HER catalytic activity. This expectation is verified by the calculations of free-energy diagram for the HER reaction paths on the Cu_5_Zr(111) facets with or without the hydroxylation of surface Zr atoms. As shown in Figure 1(f), the Cu_5_Zr-OH(111) has an energy barrier of ~0.54 eV for the rate-limiting step of H^∗^ adsorption/combination, much lower than the value of the Cu_5_Zr (~0.67 eV).

### 2.2. Preparation and Structural Characterization

**Figure 2:**
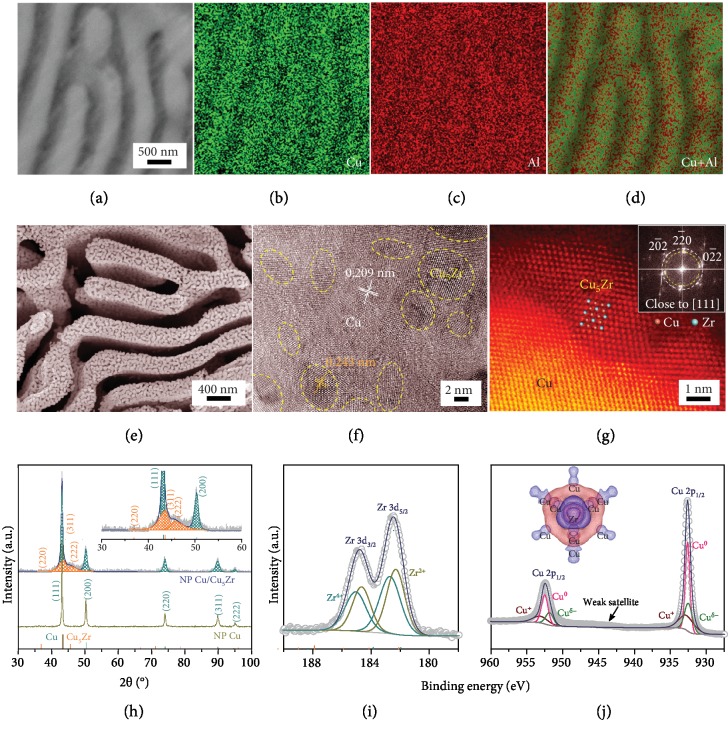
Microstructural and chemical characterization. (a) SEM image of Cu_17_Zr_3_Al_80_ precursor alloy. (b–d) The corresponding SEM-EDS mappings (Cu in green, Al in red) of Cu_17_Zr_3_Al_80_ precursor alloy. (e) Representative SEM image of dealloyed nanoporous Cu/Cu_5_Zr monolithic catalytic electrodes. (f) HRTEM image of Cu/Cu_5_Zr composite, in which Cu_5_Zr clusters anchored on Cu ligaments. (g) HAADF-STEM image of Cu/Cu_5_Zr composite, demonstrating the atomic structure of intermetallic Cu_5_Zr clusters as electroactive sites anchored on Cu ligaments. Inset: FFT patterns of Cu/Cu_5_Zr. (h) XRD patterns of as-dealloyed nanoporous Cu/Cu_5_Zr and bare NP Cu electrodes. The line patterns show reference cards 40-1322 and 04-0836 for intermetallic Cu_5_Zr compound and Cu according to JCPDS, respectively. Inset: enlarged XRD patterns of NP Cu/Cu_5_Zr. (i, j) High-resolution XPS spectra of Zr 3d (i) and Cu 2p (j). Inset: charge density difference plot of Cu_5_Zr.

With an aim at developing highly efficient and robust HER catalytic materials that should comprise sufficient available surface with high catalytic activity and have a rational structure with fast electron transfer and reliable steadiness [36], we construct self-supported monolithic NP Cu/Cu_5_Zr electrodes with a hierarchical nanoporous architecture by a facile and scalable dealloying strategy [46, 47]. Therein, both the nanoporous microstructure and the components are controlled by hereditary effect of precursor Cu_20-*x*_Zr*_x_*Al_80_ (*x* = 0, 1, 3, 5, and 20 at%) alloys during the dealloying processes [46]. Resembling the multiphase-structured Cu_20_Al_80_ alloy (supplementary [Supplementary-material supplementary-material-1]), the Cu_20-*x*_Zr*_x_*Al_80_ (*x* = 1, 3, and 5 at%) alloys are primarily composed of several-hundred-nanometer-wide *α*-Al and CuAl_2_ phases with a quasiperiodic distribution, as illustrated by a scanning electron microscope (SEM) backscattered electron image for the Cu_17_Zr_3_Al_80_ alloy (Figure 2(a)) and the corresponding energy-dispersive X-ray spectroscopy (EDS) elemental mappings (Figures 2(b)–2(d)). X-ray diffraction (XRD) characterization reveals that in addition to *α*-Al and CuAl_2_ phases, there are intermetallic Cu_5_Zr [48, 49], Al_9.83_Zr_0.17_ and Al_3_Zr clusters that are embedded in Cu-rich and *α*-Al phases, respectively, with the incorporation of Zr components (supplementary Figures [Supplementary-material supplementary-material-1] and [Supplementary-material supplementary-material-1]) [48, 49]. The multiphase structure enables the product of NP Cu/Cu_5_Zr with a bimodal nanoporous structure, as that of bare NP Cu (supplementary [Supplementary-material supplementary-material-1]), in a two-step chemical dealloying process: the rapid dissolution of the *α*-Al phase embedded with Al_9.83_Zr_0.17_ and Al_3_Zr to form large channels and the selective etching of the less-noble Al component from CuAl_2_ alloy to generate small nanopores and expose Cu_5_Zr clusters. As shown in a representative SEM image for the NP Cu/Cu_5_Zr electrode dealloyed from the precursor Cu_17_Zr_3_Al_80_ alloy (Figure 2(e)), it displays a uniform bimodal nanoporous structure consisting of large channels with ~300 nm in width and small nanopores with the diameter of ~18 nm (supplementary [Supplementary-material supplementary-material-1]). The small nanopore structure is further verified by N_2_ adsorption/desorption isotherm (supplementary [Supplementary-material supplementary-material-1]), which signifies a size distribution with maximum centered at ~18 nm (supplementary [Supplementary-material supplementary-material-1]). Figure 2(f) shows a typical high-resolution transmission electron microscope (HRTEM) image of Cu/Cu_5_Zr ligaments, where high-density intermetallic Cu_5_Zr clusters with diameter of ~3-6 nm can be identified by their distinct contrasts and lattices. Atomic resolution high-angle annual-dark-field scanning transmission electron microscope (HAADF-STEM) image viewed along the [111] zone axis illustrates that these Cu_5_Zr clusters are exposed and in situ anchored on the NP Cu skeleton with a seamless interfacial structure (Figure 2(g)), enhancing the stability of electroactive Cu_5_Zr clusters and their electron transfer with conductive Cu skeleton. The hybrid structure of NP Cu/Cu_5_Zr is further verified by the corresponding fast Fourier transform (FFT) patterns (inset of Figure 2(g)) and the XRD patterns (Figure 2(h)) with two sets of diffraction characteristics [48]. In the XRD patterns, the weak diffraction peaks at 2*θ* = 37.0, 43.7, and 45.7 are assigned to the (220), (311), and (222) planes of fcc Cu_5_Zr clusters (JCPDS 40-1322) (inset of Figure 2(h)) [48], apart from the obvious ones attributed to monometallic Cu (JCPDS 04-0836). X-ray photoelectron spectroscopy (XPS) analysis reveals the presence of Zr and Cu with an atomic ratio of 92/5, in addition to a little residual Al according to inductively coupled plasma mass spectroscopy (ICP-MS) and EDS (supplementary [Supplementary-material supplementary-material-1] and [Supplementary-material supplementary-material-1]). The deviation of Zr/Cu ratio from the initial value in the precursor alloy confirms the fact that those Al_9.83_Zr_0.17_ and Al_3_Zr are removed with the dissolution of *α*-Al phase. Because of electron donation from Zr to Cu (inset of Figure 2(j)), the Zr 3d XPS spectrum shows the chemical state of Zr^2+^ in addition to the Zr^4+^ that is due to the oxidation of surface Zr atoms (Figure 2(i)) [50]. This is in contrast with the observation of solid Cu/Cu_5_Zr bulk alloy with negligible Cu_5_Zr clusters exposed at the surface (supplementary Figures [Supplementary-material supplementary-material-1]), of which the primary chemical state of Zr^2+^ is attributed to the electron transfer from Zr to Cu (supplementary [Supplementary-material supplementary-material-1]). As a result, Cu exhibits the chemical state of Cu^*δ*-^ in addition to Cu^0^ and Cu^+^ that correspond to the Cu atoms locating at the internal and surface of Cu ligaments, respectively (Figure 2(j) and supplementary [Supplementary-material supplementary-material-1]) [34, 51]. Similar dealloying processes also take place in the precursor Cu_20-*x*_Zr*_x_*Al_80_ alloys with *x* = 1 (Cu_19_Zr_1_Al_80_) or 5 (Cu_15_Zr_5_Al_80_), which gives rise to almost the same bimodal nanoporous structure (supplementary Figures [Supplementary-material supplementary-material-1]) except for their distinct Cu/Zr compositions of 97/1 (supplementary [Supplementary-material supplementary-material-1]) and 80/16 (supplementary [Supplementary-material supplementary-material-1]), respectively. In contrast, the precursor alloy with *x* = 20 (i.e., Zr_20_Al_80_) produces NP Zr with a single-modal nanoporous structure during the chemical dealloying (supplementary [Supplementary-material supplementary-material-1]).

### 2.3. Electrochemical Characterizations

**Figure 3:**
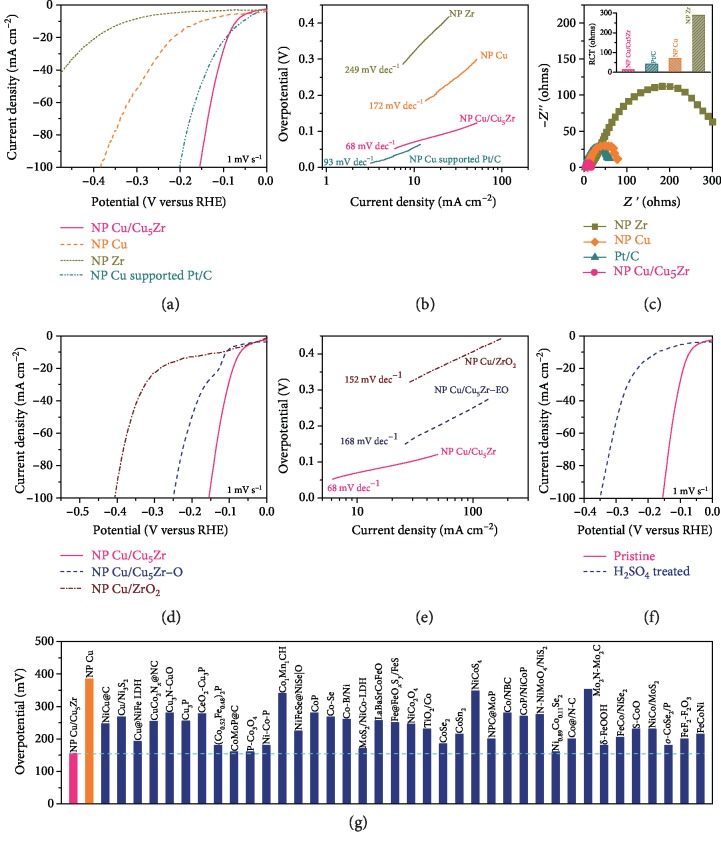
Electrochemical properties of catalysts for HER catalysis. (a, b) Polarization curves (a) and Tafel plots (b) of nanoporous Cu, Cu/Cu_5_Zr, and Zr catalyst electrodes and nanoporous Cu-supported Pt/C in 1 M KOH solution. Scan rate: 1 mV s^−1^ for nanoporous catalyst electrodes. (c) Nyquist plot comparing electrochemical impedance spectra (EIS) of nanoporous Cu, Cu/Cu_5_Zr, and Zr catalyst electrodes and nanoporous Cu-supported Pt/C. Inset: their corresponding charge transfer resistances (*R*_CT_). (d, e) Comparison of polarization curve (d) and Tafel plot (e) of the pristine NP Cu/Cu_5_Zr with those of electrooxidized NP Cu/Cu_5_Zr (NP Cu/Cu_5_Zr-EO) and NP Cu-supported ZrO_2_ nanoparticles (NP Cu/ZrO_2_) in 1 M KOH electrolyte. Scan rate: 1 mV s^−1^. (f) Polarization curves for nanoporous Cu/Cu_5_Zr before and after H_2_SO_4_ treatment. (g) Comparison of overpotentials at current density of 100 mA cm^−2^ for nanoporous Cu/Cu_5_Zr with previously reported HER catalysts in 1 M KOH electrolyte (supplementary [Supplementary-material supplementary-material-1]).

To assess the electrocatalytic HER activity of NP Cu/Cu_5_Zr, all nanoporous catalytic materials are directly used as working electrodes for electrochemical measurements in N_2_-saturated 1 M KOH aqueous electrolyte, based on a classic three-electrode configuration with a saturated calomel electrode (Hg/Hg_2_Cl_2_, SCE) and a graphite rod as the reference electrode and the counter electrode, respectively. All potentials are calibrated with respect to the RHE. Supplementary [Supplementary-material supplementary-material-1] shows the first four polarization curves of NP Cu/Cu_5_Zr electrode. In agreement with DFT simulation result, there takes place an irreversible reduction reaction of Cu hydroxide to metallic Cu at the potential of -0.17 V vs. RHE during the first HER measurement. This enables metallic surface Cu atoms to take part in the subsequent HER. In the plot of geometry area-normalized current density versus iR-corrected potential, Figure 3(a) shows the representative HER polarization curves of self-supported NP Cu/Cu_5_Zr hybrid electrode and NP Cu and Zr monometallic electrodes at a scan rate of 1 mV s^−1^, comparing with that of commercially available Pt/C, a benchmark HER catalyst, immobilized on NP Cu current collector. By virtue of the presence of Cu_5_Zr clusters, the NP Cu/Cu_5_Zr electrode exhibits remarkably enhanced HER catalytic activity, with an onset overpotential (~30 mV) much lower than the values of NP Cu (~140 mV) and Zr (~260 mV), which have either too low or too high HBE. The superior HER catalysis of NP Cu/Cu_5_Zr is also demonstrated by the ultralow Tafel slope of ~68 mV dec^−1^, compared with the values of bare NP Cu (~172 mV dec^−1^) and NP Zr (~249 mV dec^−1^) (Figure 3(b)). With the low onset overpotential and small Tafel slope due to high-density and high-activity Cu_5_Zr clusters, the NP Cu/Cu_5_Zr rapidly reaches the geometric area-normalized current density of 100 mA cm^−2^ at the potential of -155 mV versus RHE, which is higher than that of NP Cu-supported Pt/C electrode (-199 mV) although the former exhibits slightly lower activity than the latter at low overpotential range from 0 to -86 mV (Figure 3(a)). The fast kinetics of NP Cu/Cu_5_Zr electrode is further justified by electrochemical impedance spectroscopy (EIS) measurements in the frequency range of 100 kHz to 10 mHz. As shown in the Nyquist plots (Figure 3(c)), the EIS spectra of all nanoporous catalysts display characteristic semicircles in the high- and middle-frequency range, of which the diameters represent their charge transfer resistances (*R*_CT_). Based on the equivalent circuit (inset of supplementary [Supplementary-material supplementary-material-1]), the NP Cu/Cu_5_Zr is demonstrated to have the lowest *R*_CT_ value (~12 *Ω*) at the overpotential of 0.1 V among these catalysts with nanoporous architecture including the NP Cu-supported Pt/C (~41 *Ω*) (inset of Figure 3(c)), in agreement with their observations in Tafel plots (Figure 3(b)). The impressive HER catalysis results from the Cu_5_Zr clusters, on which the hydroxygenated Zr surface atoms not only adsorb strongly HO^∗^ but adjust the H^∗^ adsorption at the Cu-Cu bridge sites, essentially boosting the HER kinetics as a result of the balance between the water dissociation and the H^∗^ adsorption/desorption [52]. The unique behavior differs from the NP Cu/ZrO_2_ hybrid electrode, on which the constituent ZrO_2_ nanoparticles serve as strong HO^∗^ adsorption sites but the Cu skeleton is too weak to adsorb the H^∗^, as well as the fully electrooxidized NP Cu/Cu_5_Zr (NP Cu/Cu_5_Zr-EO), on which there are only strong HO^∗^ adsorption sites. This leads to the distinct HER activity of the pristine NP Cu/Cu_5_Zr, with the overpotential at 100 mA cm^−2^ being ~100 and ~250 mV lower than those of the NP Cu/ZrO_2_ and NP Cu/Cu_5_Zr-EO (Figure 3(d)) in addition to the exceptional Tafel slope (Figure 3(e)).

To further attest that intermetallic Cu_5_Zr clusters are indeed active sites for the HER, we investigate the NP Cu/Cu_5_Zr electrode after removing the exposed Cu_5_Zr clusters in H_2_SO_4_ solution. Supplementary [Supplementary-material supplementary-material-1] shows a typical SEM image of the H_2_SO_4_-treated Cu/Cu_5_Zr electrode, displaying almost the same nanoporous architecture as the pristine one. Nevertheless, the XRD characterization demonstrates that most of the Cu_5_Zr are removed except for a few clusters imbedded in the Cu ligaments (supplementary [Supplementary-material supplementary-material-1]), as confirmed by the Cu/Zr/Al component change of 98.9/0.4/0.7 (supplementary [Supplementary-material supplementary-material-1]) as well as the Zr 3d and Cu 2p XPS spectra with little amount of Cu^*δ*-^ and Zr^2+^ (supplementary Figures [Supplementary-material supplementary-material-1]).  Figure 3(f) compares the HER polarization curve of the H_2_SO_4_-treated NP Cu/Cu_5_Zr with that of the pristine one. Owing to the absence of Cu_5_Zr clusters at the surface, the H_2_SO_4_-treated NP Cu/Cu_5_Zr exhibits remarkably degraded HER activity, with the overpotential at the current density of 100 mA cm^−2^ increasing to ~350 mV, which implies the indispensable role of Cu_5_Zr clusters in achieving superior HER performance of NP Cu/Cu_5_Zr electrodes. This is also reflected by the linear dependence of the overpotentials at 100 mA cm^−2^ on the surface areas, which are adjusted by controlling the dealloying time (supplementary Figures [Supplementary-material supplementary-material-1]) or precursor alloy components (supplementary [Supplementary-material supplementary-material-1]). Although there is always residual Al component in the ligaments, it is not expected to significantly influence the HER catalytic activity of NP Cu/Cu_5_Zr. As shown in supplementary [Supplementary-material supplementary-material-1], the NP Cu/Cu_5_Zr electrodes with different residual amount of Al exhibit almost the same specific activities at the overpotential of 100 mV. To evaluate quantitatively the HER activity of Cu_5_Zr clusters, here we approximately estimate the specific current density (*j*_s,Cu_5_Zr_) of the electroactive Cu_5_Zr clusters according to the equation [53] *j*_s,Cu_5_Zr_ = (*j*_Cu/Cu_5_Zr_*A*_Cu/Cu_5_Zr_ − *j*_s,Cu_*A*_s,Cu_)/*A*_s,Cu_5_Zr_, where *j*_Cu/Cu_5_Zr_ and *j*_s,Cu_ are the specific current densities of NP Cu/Cu_5_Zr and the constituent Cu surface, *A*_Cu/Cu_5_Zr_ = *A*_s,Cu_ + *A*_s,Cu_5_Zr_ with *A*_s,Cu_ and *A*_s,Cu_5_Zr_ being the specific surface areas of Cu and Cu_5_Zr components in the NP Cu/Cu_5_Zr, respectively. Considering that the NP Cu/Cu_5_Zr and bare NP Cu electrodes have almost the same nanoporous architectures with primary (111) surface (Figure 2(e) and supplementary [Supplementary-material supplementary-material-1]), the value of *j*_s,Cu_ refers to that of NP Cu while the *A*_s,Cu_ is calculated through the broad anodic peak between 0.5 and 0.71 V corresponding to the formation of Cu_2_O with a charge density of 360 *μ*C cm^−2^ (supplementary [Supplementary-material supplementary-material-1]) [25]. With an assumption that the Zr^2+^ corresponds to the surface Zr atom, *A*_s,Cu_5_Zr_ is evaluated according to *A*_s,Cu_5_Zr_ = *cA*_s,Cu_ with *c* being the surface Zr^2+^/Cu component ratio. At the potential of -200 mV versus RHE, *j*_s,Cu_5_Zr_ reaches ~3.1 mA cm^−2^_SSA_, ~300-fold enhancement relative to Cu. This enlists NP Cu/Cu_5_Zr to outstand among the best nonprecious metal-based HER catalysts, including alloys and compounds, as shown in Figure 3(g) and supplementary [Supplementary-material supplementary-material-1] for the overpotential comparisons at various current densities in 1 M KOH electrolyte.

**Figure 4:**
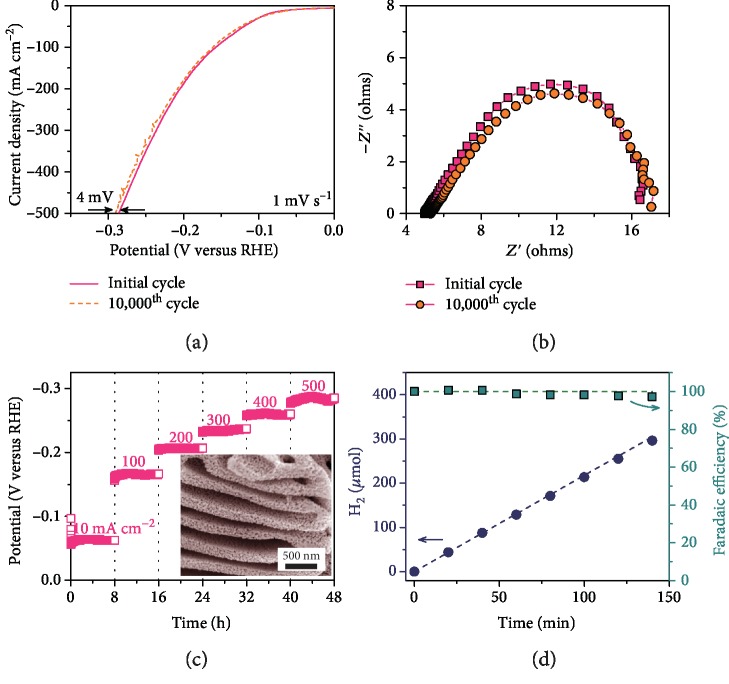
HER performance of nanoporous Cu/Cu_5_Zr electrode in alkaline electrolyte. (a, b) Polarization curves (a) and the corresponding Nyquist plots of EIS spectra (b) for nanoporous Cu/Cu_5_Zr catalyst electrode before and after 10,000 potential cycles. (c) Long-term stability tests of nanoporous Cu/Cu_5_Zr at different current densities. Inset: typical SEM image of nanoporous Cu/Cu_5_Zr electrode after stability measurement. (d) The production and Faradaic efficiency of theoretical hydrogen generation (dash line) and practical hydrogen (dot).

As a consequence of the unique monolithic and steady architecture, all NP Cu/Cu_5_Zr electrodes with different Cu/Zr/Al compositions can reach the current density of as high as 500 mA cm^−2^ at low overpotentials in 1 M KOH aqueous solution (supplementary [Supplementary-material supplementary-material-1]). It takes the NP Cu_92_Zr_5_Al_3_ electrode only an overpotential of -280 mV, lower than the values of NP Cu_97_Zr_1_Al_2_ and Cu_80_Zr_16_Al_4_ that have either less electroactive Cu_5_Zr clusters or smaller specific surface areas (supplementary [Supplementary-material supplementary-material-1]). To our knowledge, this is the lowest overpotential reported in literature (supplementary [Supplementary-material supplementary-material-1]). Furthermore, the NP Cu/Cu_5_Zr electrodes exhibit exceptional structural and electrochemical durability in the accelerated stability tests, which are carried out by the continuous potential cycling between -0.2 and 0.1 V versus RHE at a scan rate of 100 mV s^−1^ in 1 M KOH solution. As shown in supplementary [Supplementary-material supplementary-material-1], the NP Cu/Cu_5_Zr electrode not only maintains the nanoporous architecture but keeps the constitutes of Cu ligaments and Cu_5_Zr clusters with almost the same chemical states. Although Al thermodynamically tends to dissolve in basic solution (the standard dissolution potential of -1.66 V versus RHE for Al to Al^3+^), ICP measurement indicates that there is no evident change of Al component because of the protection of Cu surface layer. As a consequence, the HER polarization curve after 10,000 cycles only shifts negatively by 4 mV relative to the initial one, which leads to ~2% decay of current density at the overpotential of ~280 mV (Figure 4(a)) probably due to a slight increase in charge transfer resistance (Figure 4(b)). To simulate the industrial product of H_2_ at large current density, we perform a galvanostatic stability test at various current densities from 10 to 500 mA cm^−2^ in 1 M KOH electrolyte (Figure 4(c)). When performing at each current density for 8 h, the NP Cu/Cu_5_Zr electrode exhibits stable potentials, indicating the outstanding durability. Even at the current density of as high as 500 mA cm^−2^, the NP Cu/Cu_5_Zr electrode can withstand the violent H_2_ gas evolution to maintain the original nanoporous structure in the long-term durability measurement (inset of Figure 4(c)). The impressive catalytic stability can be attributed to the electrochemical stability of Cu_5_Zr clusters and the robustness of nanoporous Cu structure for hydrogen bubble release. Moreover, the practical hydrogen production rate in Figure 4(d) agrees well with the theoretical value, revealing that the Faradaic efficiency reaches approximately 100%.

## 3. Conclusions

In summary, we have developed monolithic and hierarchical nanoporous Cu/Cu_5_Zr hybrid electrodes as low-cost and robust catalytic materials towards the HER in alkaline environments. As a consequence of evident electron donation from Zr to Cu triggered by their large electronegative difference, the zirconium atoms essentially activate their nearby Cu-Cu bridge sites. While the hydroxylation of surface Zr atoms facilitates the HO^∗^ adsorption and adjusts an appropriate hydrogen-binding energy conducive to the adsorption/desorption of reactive hydrogen intermediates, the constituent Cu_5_Zr intermetallic clusters serve as stable HER catalysts with exceptionally high activity of ~3.1 mA cm^−2^ (~300-fold enhancement relative to bare Cu) at the potential of -200 mV versus RHE. Associated with the unique architecture featured with steady hierarchical nanoporous Cu current collector that not only facilitates electron transfer to the constituent Cu_5_Zr clusters but also endures the violent gas evolution, the NP Cu/Cu_5_Zr electrode exhibits superior HER electrocatalytic activity and durability in 1 M KOH solution relative to their corresponding monometallic catalysts with similar architecture, such as the NP Cu and Zr electrodes. More impressively, the NP Cu/Cu_5_Zr only takes a low potential of -280 mV versus RHE to achieve the current density of as high as 500 mA cm^−2^, outperforming the state-of-the-art nonprecious HER catalysts. The outstanding electrochemical properties enable the NP Cu/Cu_5_Zr to show genuine potential as an attractive alternative to precious metal-based catalysts for large-scale and efficient hydrogen production through water electrolysis.

## 4. Methods

### 4.1. Fabrication of Self-Supported Nanoporous Catalyst Electrodes

Catalyst electrodes with a nanoporous structure were fabricated by a facile alloying/dealloying procedure. Precursor Cu_20-*x*_Zr*_x_*Al_80_ (*x* = 0, 1, 3, 5, and 20 at%) alloys were produced by arc melting pure Cu, Zr, and Al under an argon atmosphere. After cutting into thin sheets with thickness of ~400 *μ*m and further polishing by sandpapers (600, 1200, and 2000 Cw), nanoporous catalyst electrodes were prepared by chemical dealloying in a N_2_-purged 6 M KOH aqueous electrolyte at 70°C until there is no gas to be produced. To identify the electroactive sites that enhanced the Zr atoms, the nanoporous Cu/Cu_5_Zr catalyst electrode was further treated in a N_2_-purged 0.5 M H_2_SO_4_ aqueous electrolyte at 70°C to remove surface Zr atoms. All these catalysts were rinsed in ultrapure water (18 M*Ω*) for multiple times to remove substance in nanoporous structure. Commercially available Pt/C catalyst with weight of 1.5 mg (20 wt%, Johnson Matthey) was mixed into a Nafion (0.05 wt%, Sigma-Aldrich) solution containing isopropanol (20%) and water (80%) to form Pt/C ink under rigorous sonication. 100 *μ*L Pt/C ink was drop-cast onto NP Cu electrode (2 mm × 5 mm × 0.4 mm) to prepare the Pt/C catalyst electrode for electrochemical measurements.

### 4.2. Structural Characterization

Field-emission scanning electron microscope (JSM-6700F, JEOL, 15 keV) equipped with X-ray energy-dispersive spectroscopy (EDS) was employed to characterize microstructure and chemical composition of nanoporous catalysts. High-resolution transmission electron microscopy (HRTEM) and scanning transmission electron microscopy (STEM) characterizations were performed on a field-emission transmission electron microscope (JEM-2100F, JEOL, 200 keV) and a field-emission transition electron microscope (JEM-ARM200CF, JEOL) operated at 200 keV and equipped with double spherical-aberration correctors for both condenser and objective lens, respectively. X-ray diffraction patterns of all nanoporous catalyst electrodes were collected from a D/max2500pc diffractometer with a monochromated Cu K*_α_* radiation. X-ray photoelectron spectroscopy (XPS) analysis was conducted on a Thermo ECSALAB 250 with an Al anode. Charging effects were compensated by shifting binding energies based on the adventitious C 1s peak (284.8 eV). Atomic ratio of elements was analyzed using ICP-MS (Thermo electron). N_2_ adsorption/desorption isotherms were collected at 77 K by a Micromeritics (ASAP 2020 Plus) system.

### 4.3. Electrochemical Measurements

All nanoporous catalyst electrodes were directly used as working electrodes for electrochemical measurements, which were performed in a classical three-electrode setup with a graphite rod as the counter electrode and a saturated calomel electrode (Hg/Hg_2_Cl_2_, SCE) as the reference electrode. The HER polarization curves of nanoporous catalyst electrodes were collected at a scan rate of 1 mV s^−1^ in a N_2_-saturated 1 M KOH aqueous solution at room temperature. The reference electrode was calibrated to the reversible hydrogen potential (RHE). Electrochemical impedance spectroscopy (EIS) analysis was performed at various overpotentials with frequency 0.01 to 100,000 Hz with 5 mV amplitude. To evaluate the electrochemical surface area (ECSA), cyclic voltammograms (CVs) were collected from 0.5 to 1.65 V (versus RHE) at a scan rate of 20 mV s^−1^. The HER stability tests of nanoporous Cu/Cu_5_Zr electrode were carried out at the current densities of 10, 100, 200, 300, 400, and 500 mA cm^−2^ in 1 M KOH solution, respectively, for 8 h. In addition, a potential cycling in a window of -0.2 and 0.1 V was also performed for 30,000 cycles with a scan rate of 100 mV s^−1^ in a N_2_-saturated 1 M KOH solution. The hydrogen production via the HER experiments were further conducted at the current density of 20 mA cm^−2^ for NP Cu/Cu_5_Zr electrode (0.35 cm^−2^) in a typical H-type cell separated by a proton exchange membrane, and the generated gas was analyzed by gas chromatography (GC-2014).

### 4.4. DFT Calculation

All spin-unrestricted DFT calculations were carried out using the Vienna Ab initio Simulation Package (VASP) with the Perdew, Burke, and Ernzerhof (PBE) functional. The projector augmented wave (PAW) method was adopted to describe the electron-ion interactions. According to our careful convergence tests, the cutoff of plane wave basis sets was set to 400 eV. A Monkhorst-Pack grid of 4 × 4 × 1 and 12 × 12 × 3 k-points were used for geometry optimization and electronic structure calculations, respectively. A first-order Methfessel-Paxton smearing of 0.2 eV was applied to accelerate electronic convergence for geometry optimization. The electronic structure and geometry optimization convergence tolerance were 10^−5^ eV, 0.005 eV Å^−1^, respectively. The transition states were obtained using the Climbing Image Nudged Elastic Band (CI-NEB) method. The convergence tolerance of NEB image was 0.05 eV Å^−1^. The Cu(111), Cu_5_Zr(111), and single-Zr-atom–doped Cu(111) and Zr(111) were modeled by using five layers, the bottom two layers were fixed while others were fully relaxed. A vacuum layer of 15 Å was used to avoid the unwanted interaction between the slab and its period images. The optimized structures and the charge density difference plots were illustrated with VESTA software.

The reaction free energy (Δ*G*) was calculated according to Δ*G* = Δ*E* + Δ*E*_ZPE_ − *T*Δ*S*, where Δ*E* is the reaction energy, Δ*E*_ZPE_ is the zero-point energy, *T* is the absolute temperature, and Δ*S* is the change of entropy. Δ*E*_ZPE_ = 1/2∫*F*(*ω*)*ħωdω*, where *ħ* is the Planck constant, *F*(*ω*) is the phonon density of states. Δ*S*_H_ is considered as −1/2 *S*^0^_H2_ due to the fact that the vibrational entropy in the adsorbed state is small meaning, where *S*^0^_H2_ is the entropy of H_2_ in the gas phase at standard conditions. In addition, the adsorption energy (*E*_M‐H_, H denoting the corresponding species) was determined by the equation *E*_M‐H_ = *E*_T_ − *E*_M_ − *E*_H_, where *E*_T_ is the total energy of the catalyst with species, *E*_M_ and *E*_H_ are the energies of the isolated catalyst and the species, respectively.

## Supplementary Materials

Supplementary Materials Figure S1: relative energies of HO^∗^ adsorptions on Cu_5_Zr at different potentials of –1.80, –1.31, 0, 0.61, and 1.00 V versus RHE. Figure S2: (a, b) atomic schematic showing Cu_5_Zr with hydrogen atom adsorption at five possible sites before (a) and after optimization (b). (c, d) Atomic schematic illustrating Cu_5_Zr-OH with hydrogen atom adsorption at four possible sites before (c) and after optimization (d). Figure S3: XRD patterns of precursor Cu_20–*x*_Zr*_x_*Al_80_ alloys with *x* = 0 (a), 1 (b), 3 (c), and 5 at% (d). The line patterns show reference cards 25-0012 for CuAl_2_ (violet line), 65-2869 for *α*-Al (dark yellow line), 40-1322 for Cu_5_Zr (pink line), 26-0041 for Al_9.83_Zr_0.17_ (orange line), and 48-1385 for Al_3_Zr (olive line) according to JCPDS. Figure S4: typical SEM image of precursor Cu_17_Zr_3_Al_80_ alloy and the corresponding EDS elemental mapping. Figure S5: typical SEM image of bare NP Cu that is fabricated by chemical dealloying Cu_20_Al_80_. It displays a bimodal and bicontinuous nanoporous architecture. Figure S6: distributions of small and large pore sizes for the NP Cu/Cu_5_Zr electrodes dealloyed from Cu_17_Zr_3_Al_80_ alloy. Figure S7: N_2_ adsorption/desorption characterization for the NP Cu/Cu_5_Zr electrode dealloyed from Cu_17_Zr_3_Al_80_ alloy. (a) The N_2_ adsorption/desorption isotherm from NP Cu/Cu_5_Zr with BET specific area of 45.4 m^2^/g. (b) Small pore size distribution of NP Cu/Cu_5_Zr. Figure S8: EDS spectrum of NP Cu/Cu_5_Zr electrode that is fabricated by chemical dealloying Cu_17_Zr_3_Al_80_ in 6 M KOH solution. Figure S9: (a, b) XRD patterns (a) and EDS spectrum (b) of solid Cu/Cu_5_Zr bulk alloy with the Cu/Zr component of 94.85/5.15, the same as that of NP Cu/Cu_5_Zr electrode. (c, d) High-resolution Zr 3d (c) and Cu 2p (d) XPS spectra for the solid Cu/Cu_5_Zr bulk electrode. Figure S10: typical SEM image (a) and EDS spectrum (b) of NP Cu/Cu_5_Zr electrode that is fabricated by chemical dealloying Cu_19_Zr_1_Al_80_ in 6 M KOH solution. Figure S11: typical SEM image (a) and EDS spectrum (b) of NP Cu/Cu_5_Zr electrode that is fabricated by chemical dealloying Cu_15_Zr_5_Al_80_ in 6 M KOH solution. Figure S12: typical SEM image of NP Zr that is fabricated by chemical dealloying Zr_20_Al_80_. It displays a single-modal and bicontinuous nanoporous architecture. Figure S13: the first four polarization curves of NP Cu/Cu_5_Zr electrode in 1 M KOH with scan rates of 5 mV s-1. The high-valence copper is reduced to metallic copper at -0.17 V. Figure S14: comparison of charge transfer resistances (RCT) and intrinsic resistance (Rs) for NP Cu/Cu_5_Zr, NP Cu-supported Pt/C, NP Cu, and NP Zr. Inset: equivalent circuit according to which their EIS spectra are analyzed. Figure S15: (a) typical SEM image of NP Cu/Cu_5_Zr electrode after H_2_SO_4_ treatment. (b) Comparison of XRD patterns of NP Cu/Cu_5_Zr before and after H_2_SO_4_ treatment. (c) EDS spectrum of NP Cu/Cu_5_Zr electrode after H_2_SO_4_ treatment. (d, e) Zr 3d (d) and Cu 2p (e) XPS spectra of NP Cu/Cu_5_Zr after H_2_SO_4_ treatment. Figure S16: (a, b) typical HER polarization curves (a) and CV curves (b) of NP Cu/Cu_5_Zr electrodes that are fabricated by chemical dealloying precursor Cu_17_Zr_3_Al_80_ alloy for 3 h, 5 h, and 7 h at room temperature in 6 M KOH solution, respectively. Therein, the Al component changes from 8.7% to 3.4%. (c) Overpotential at 100 mA cm^–2^ for the NP Cu/Cu_5_Zr electrodes as a function of specific surface area. (d) Electrochemical surface area-normalized specific activity of the NP Cu/Cu_5_Zr electrodes that are prepared by chemical dealloying for 3, 5, and 7 h, respectively. Figure S17: (a) comparison of polarization curves of NP Cu/Cu_5_Zr electrodes with different Cu/Zr/Al compositions, which are fabricated from precursor Cu_20-*x*_Zr*_x_*Al_80_ alloys with *x* = 1, 3, and 5. Scan rate of 1 mV s^–1^. Electrolyte: 1 M KOH. (b) The overpotential at the current density of 500 mA cm^–2^ for NP Cu/Cu_5_Zr as a function of specific surface area. Figure S18: typical CV curves of bare NP Cu (a) and NP Cu/Cu_5_Zr with various Cu/Zr/Al components (b–d) that are fabricated by chemical dealloying Cu_20_Al_80_ and Cu_20-*x*_Zr*_x_*Al_80_ (*x* = 1, 3, and 5 at%), respectively. Scan rate: 20 mV s^–1^. Figure S19: (a, b) typical SEM image (a) and XRD patterns (b) of NP Cu/Cu_5_Zr electrode after potential cycling measurement (10,000 cycles). (c, d) Zr 3d (c) and Cu 2p (d) XPS spectra of NP Cu/Cu_5_Zr after 10,000 cycles. Table S1: the HBE values at the possible adsorption sites on the Cu_5_Zr and Cu_5_Zr-OH illustrated in Figure S2. Table S2: chemical compositions of NP Cu/Cu_5_Zr dealloyed from different precursors, which are determined by ICP-MS, EDS, and XPS analysis. Table S3: comparisons of the catalytic activity of NP Cu/Cu_5_Zr electrocatalytic materials in alkaline medium (pH = 14) with representative noble metal-free ones reported previously.
