# Supplementary material for: Intermetallic Cu5Zr Clusters Anchored on Hierarchical Nanoporous Copper as Efficient Catalysts for Hydrogen Evolution Reaction
Source: Research (Wash D C). 2020 Feb 20;2020:2987234. doi: 10.34133/2020/2987234 (PMC7053377; doi:10.34133/2020/2987234)
Supplement: Supplementary Materials — Figure S1: relative energies of HO∗ adsorptions on Cu5Zr at different potentials of –1.80, –1.31, 0, 0.61, and 1.00 V versus RHE. Figure S2: (a, b) atomic schematic showing Cu5Zr with hydrogen atom adsorption at five possible sites before (a) and after optimization (b). (c, d) Atomic schematic illustrating Cu5Zr-OH with hydrogen atom adsorption at four possible sites before (c) and after optimization (d). Figure S3: XRD patterns of precursor Cu20–xZrxAl80 alloys with x = 0 (a), 1 (b), 3 (c), and 5 at% (d). The line patterns show reference cards 25-0012 for CuAl2 (violet line), 65-2869 for α-Al (dark yellow line), 40-1322 for Cu5Zr (pink line), 26-0041 for Al9.83Zr0.17 (orange line), and 48-1385 for Al3Zr (olive line) according to JCPDS. Figure S4: typical SEM image of precursor Cu17Zr3Al80 alloy and the corresponding EDS elemental mapping. Figure S5: typical SEM image of bare NP Cu that is fabricated by chemical dealloying Cu20Al80. It displays a bimodal and bicontinuous nanoporous architecture. Figure S6: distributions of small and large pore sizes for the NP Cu/Cu5Zr electrodes dealloyed from Cu17Zr3Al80 alloy. Figure S7: N2 adsorption/desorption characterization for the NP Cu/Cu5Zr electrode dealloyed from Cu17Zr3Al80 alloy. (a) The N2 adsorption/desorption isotherm from NP Cu/Cu5Zr with BET specific area of 45.4 m2/g. (b) Small pore size distribution of NP Cu/Cu5Zr. Figure S8: EDS spectrum of NP Cu/Cu5Zr electrode that is fabricated by chemical dealloying Cu17Zr3Al80 in 6 M KOH solution. Figure S9: (a, b) XRD patterns (a) and EDS spectrum (b) of solid Cu/Cu5Zr bulk alloy with the Cu/Zr component of 94.85/5.15, the same as that of NP Cu/Cu5Zr electrode. (c, d) High-resolution Zr 3d (c) and Cu 2p (d) XPS spectra for the solid Cu/Cu5Zr bulk electrode. Figure S10: typical SEM image (a) and EDS spectrum (b) of NP Cu/Cu5Zr electrode that is fabricated by chemical dealloying Cu19Zr1Al80 in 6 M KOH solution. Figure S11: typical SEM image (a) and EDS spectrum (b) of NP C [file 2987234.f1.docx]

**Supplementary information**

**for**

**Intermetallic Cu_5_Zr clusters anchored on hierarchical nanoporous copper as efficient catalysts for hydrogen evolution reaction**

Hang Shi^1✝^, Yi-Tong Zhou^1✝^, Rui-Qi Yao^1^, Wu-Bin Wan^1^, Qing-Hua Zhang^2^, Lin Gu^2^, Zi Wen^1^, Xing-You Lang^1^*, Qing Jiang^1^*

*^1^ Key Laboratory of Automobile Materials (Jilin University), Ministry of Education, and School of Materials Science and Engineering, Jilin University, Changchun 130022, China*

*^2^ Beijing National Laboratory for Condensed Matter Physics, The Institute of Physics, Chinese Academy of Sciences, Beijing 100190, China.*


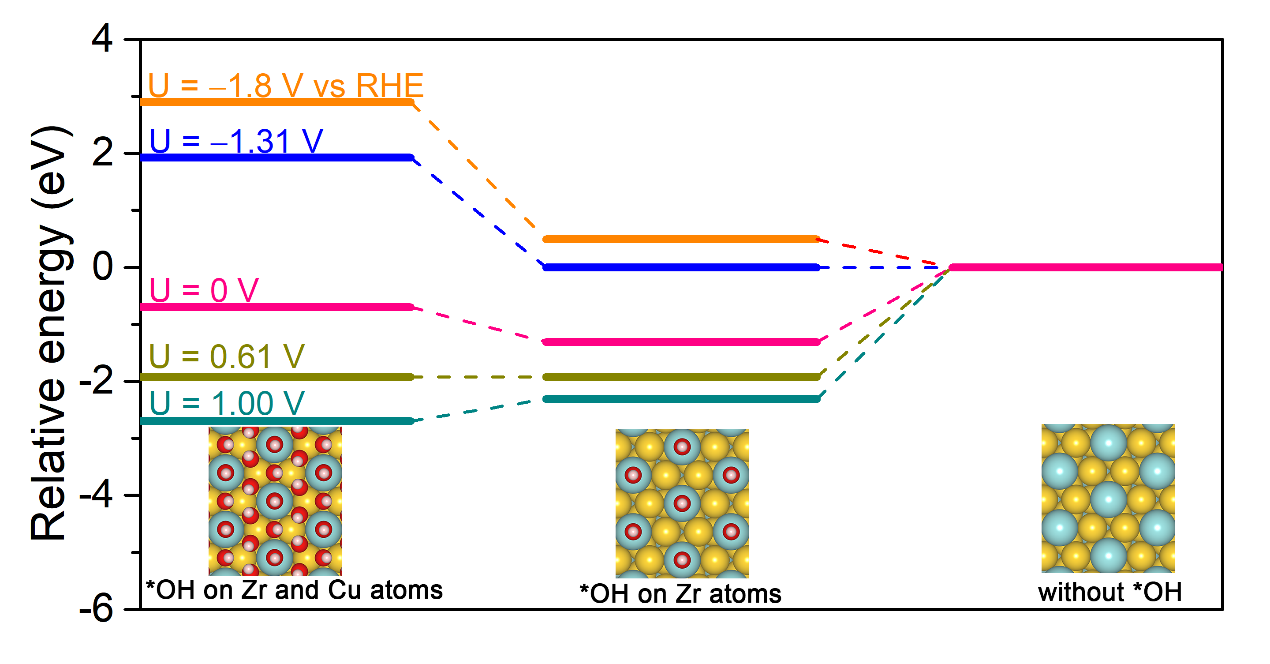


**Figure S1**. Relative energies of HO* adsorptions on Cu_5_Zr at different potentials of −1.80, −1.31, 0, 0.61 and 1.00 V versus RHE.


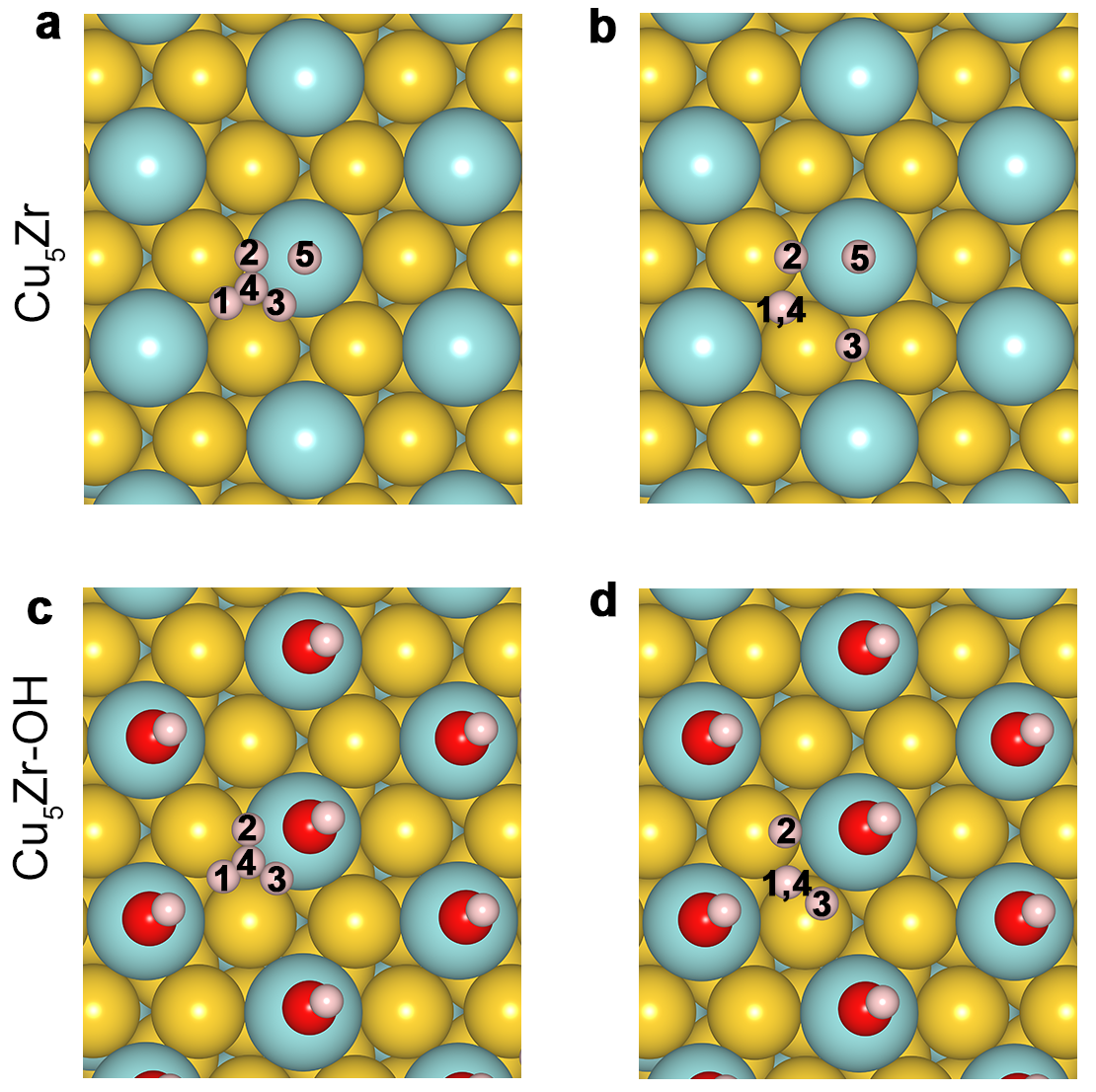


**Figure S2**. **a**,**b** Atomic schematic showing Cu_5_Zr with hydrogen atom adsorption at five possible sites before (a) and after optimization (b). **c**,**d**, Atomic schematic illustrating Cu_5_Zr-OH with hydrogen atom adsorption at four possible sites before (c) and after optimization (d).

**
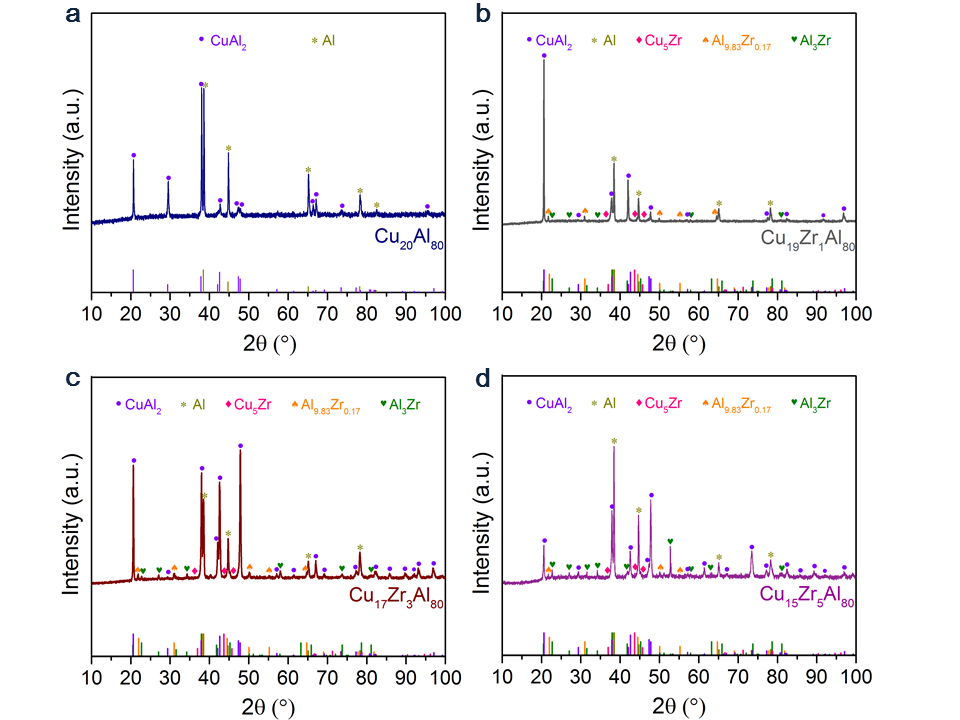
**

**Figure S3**. XRD patterns of precursor Cu_20−_*_x_*Zr*_x_*Al_80_ alloys with *x* = 0 (a), 1 (b), 3 (c) and 5 at% (d). The line patterns show reference cards 25-0012 for CuAl_2_ (violet line), 65-2869 for α-Al (dark yellow line), 40-1322 for Cu_5_Zr (pink line), 26-0041 for Al_9.83_Zr_0.17_ (orange line), and 48-1385 for Al_3_Zr (olive line) according to JCPDS.


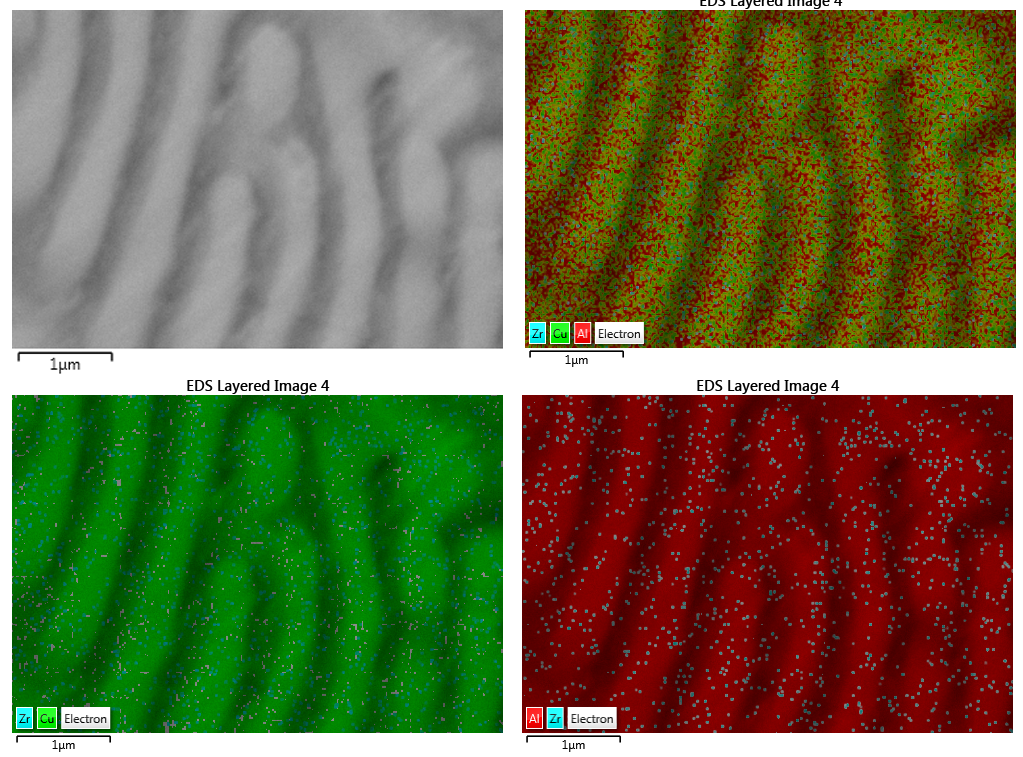


**Figure S4**. Typical SEM image of precursor Cu_17_Zr_3_Al_80_ alloy and the corresponding EDS elemental mapping.


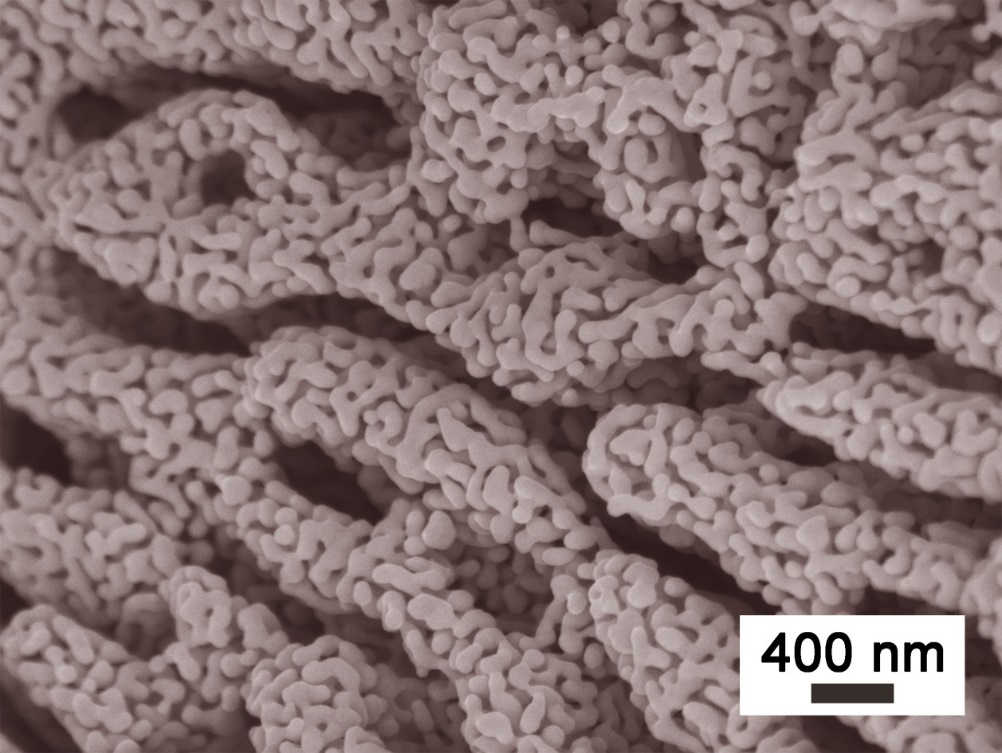


**Figure S5**. Typical SEM image of bare NP Cu that is fabricated by chemical dealloying Cu_20_Al_80_. It displays a bimodal and bicontinuous nanoporous architecture.





**Figure S6**. Distributions of small and large pore sizes for the NP Cu/Cu_5_Zr electrodes dealloyed from Cu_17_Zr_3_Al_80_ alloy.


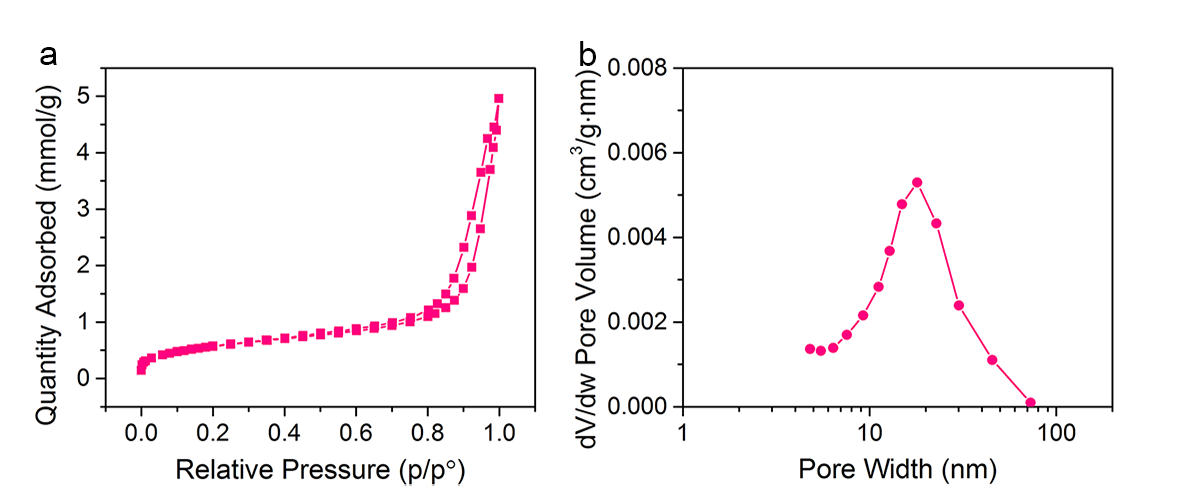


**Figure S7**. N_2_ adsorption/desorption characterization for the NP Cu/Cu_5_Zr electrode dealloyed from Cu_17_Zr_3_Al_80_ alloy. **a**, The N_2_ adsorption/desorption isotherm from NP Cu/Cu_5_Zr with BET specific area of 45.4 m^2^/g. **b**, Small pore size distribution of NP Cu/Cu_5_Zr.


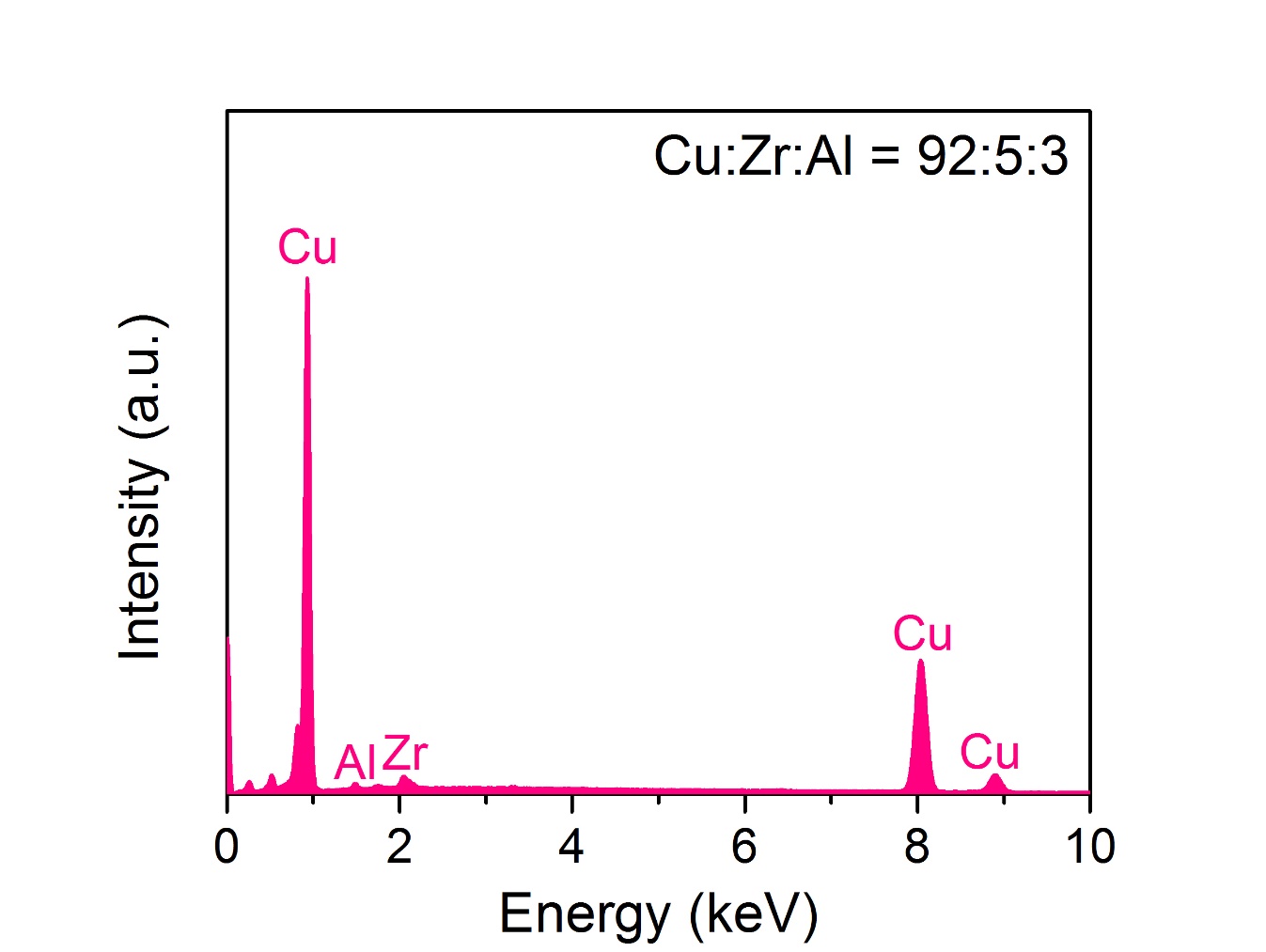


**Figure S8**. EDS spectrum of NP Cu/Cu_5_Zr electrode that is fabricated by chemical dealloying Cu_17_Zr_3_Al_80_ in 6 M KOH solution.


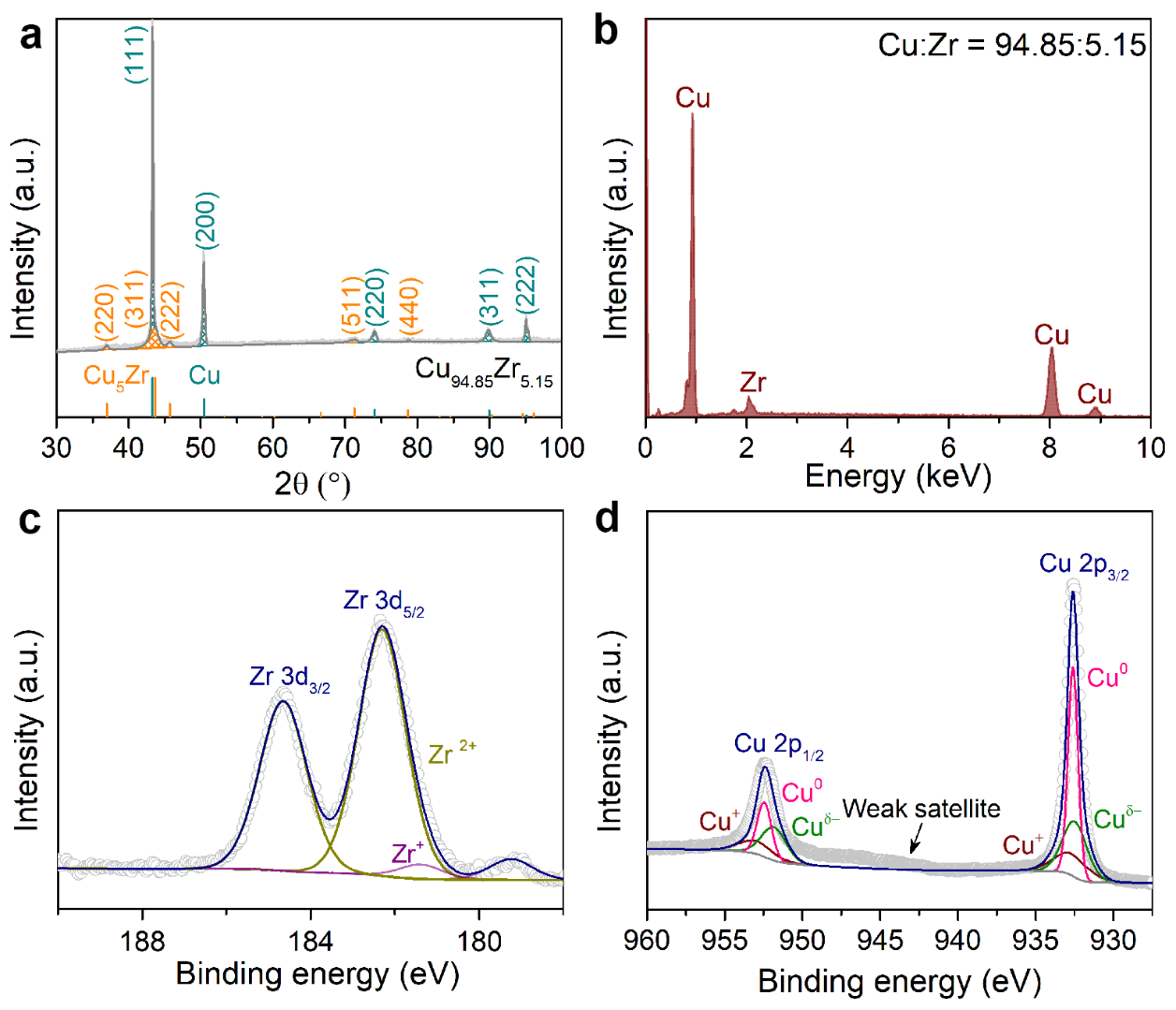


**Figure S9**. **a**, **b**, XRD patterns (a) and EDS spectrum (b) of solid Cu/Cu_5_Zr bulk alloy with the Cu/Zr component of 94.85/5.15, the same as that of NP Cu/Cu_5_Zr electrode. **c**, **d**, High-resolution Zr 3d (c) and Cu 2p (d) XPS spectra for the solid Cu/Cu_5_Zr bulk electrode.


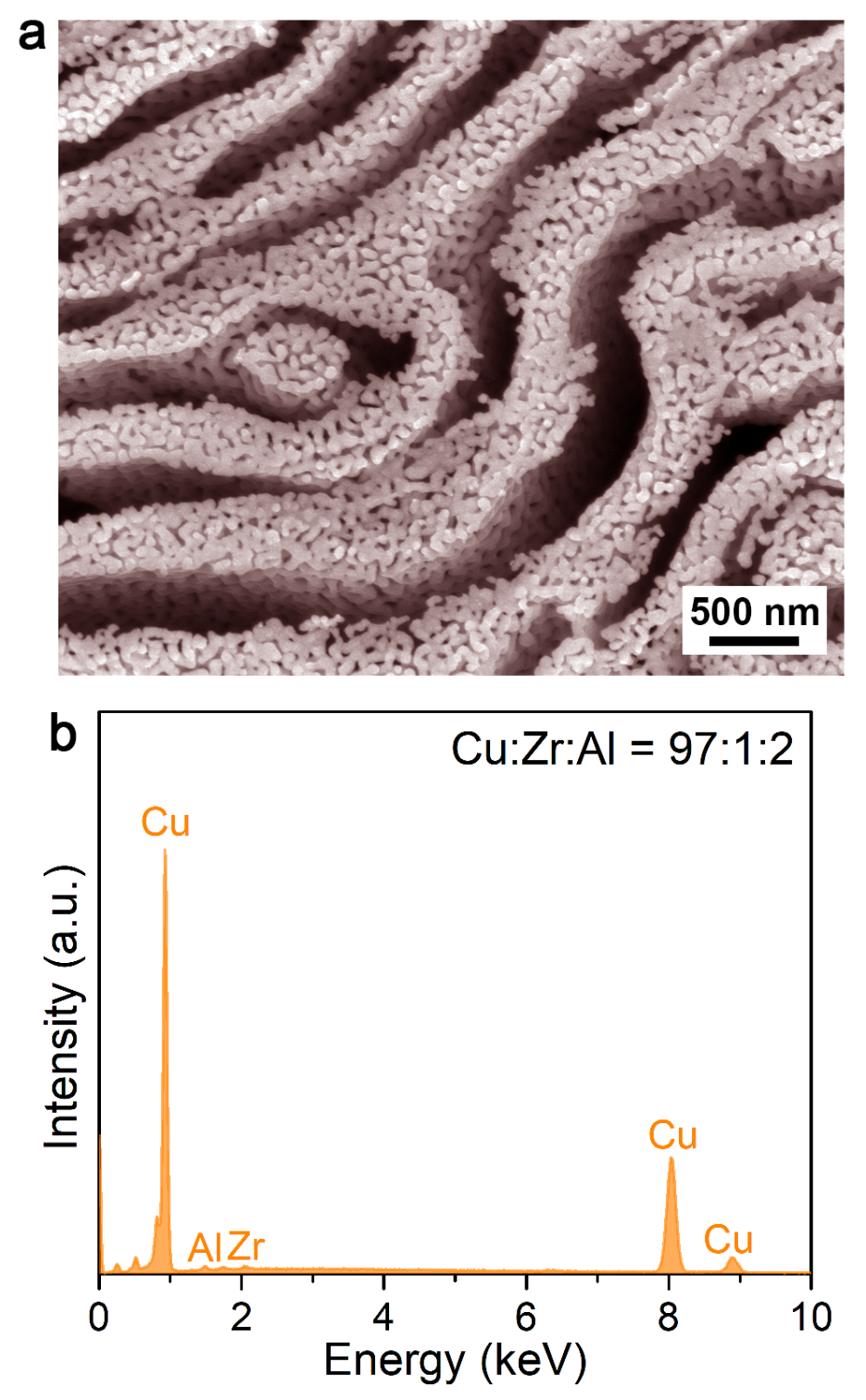


**Figure S10**. Typical SEM image (a) and EDS spectrum (b) of NP Cu/Cu_5_Zr electrode that is fabricated by chemical dealloying Cu_19_Zr_1_Al_80_ in 6 M KOH solution.


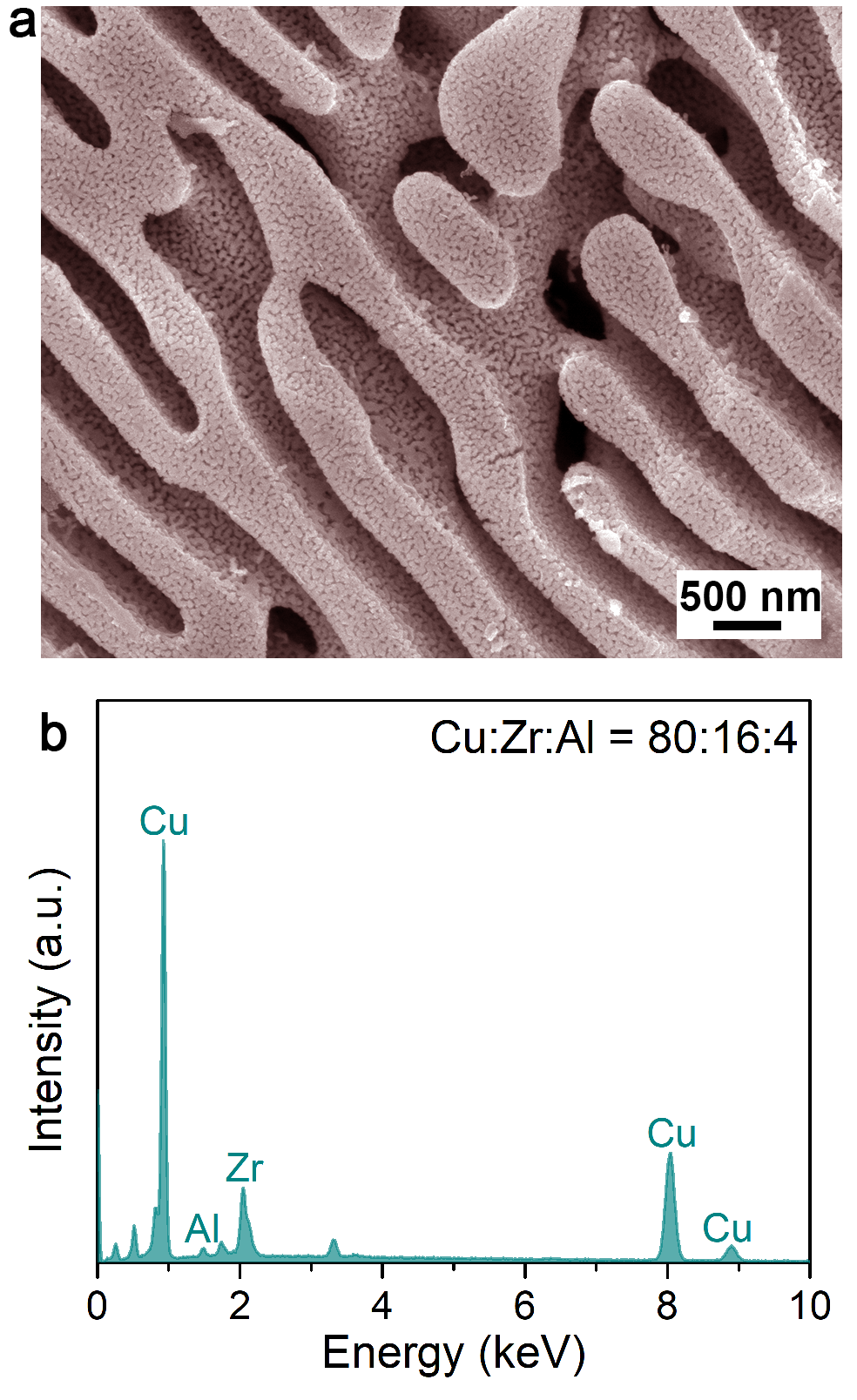


**Figure S11**. Typical SEM image (a) and EDS spectrum (b) of NP Cu/Cu_5_Zr electrode that is fabricated by chemical dealloying Cu_15_Zr_5_Al_80_ in 6 M KOH solution.


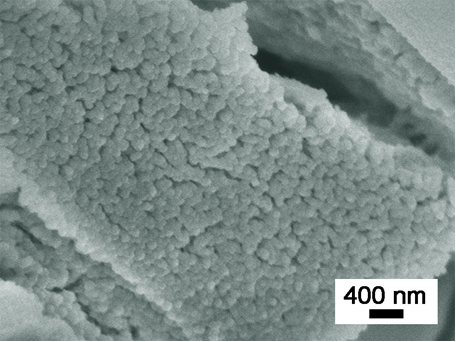


**Figure S12**. Typical SEM image of NP Zr that is fabricated by chemical dealloying Zr_20_Al_80_. It displays a single-modal and bicontinuous nanoporous architecture.





**Figure S13**. The first four polarization curves of NP Cu/Cu_5_Zr electrode in 1 M KOH with scan rates of 5 mV s^-1^. The high-valence copper is reduced to metallic copper at -0.17 V.





**Figure S14**. Comparison of charge-transfer resistances (*R*_CT_) and intrinsic resistance (*R*_s_) for NP Cu/Cu5Zr, NP Cu supported Pt/C, NP Cu and NP Zr. Inset: equivalent circuit according to which their EIS spectra are analysed.


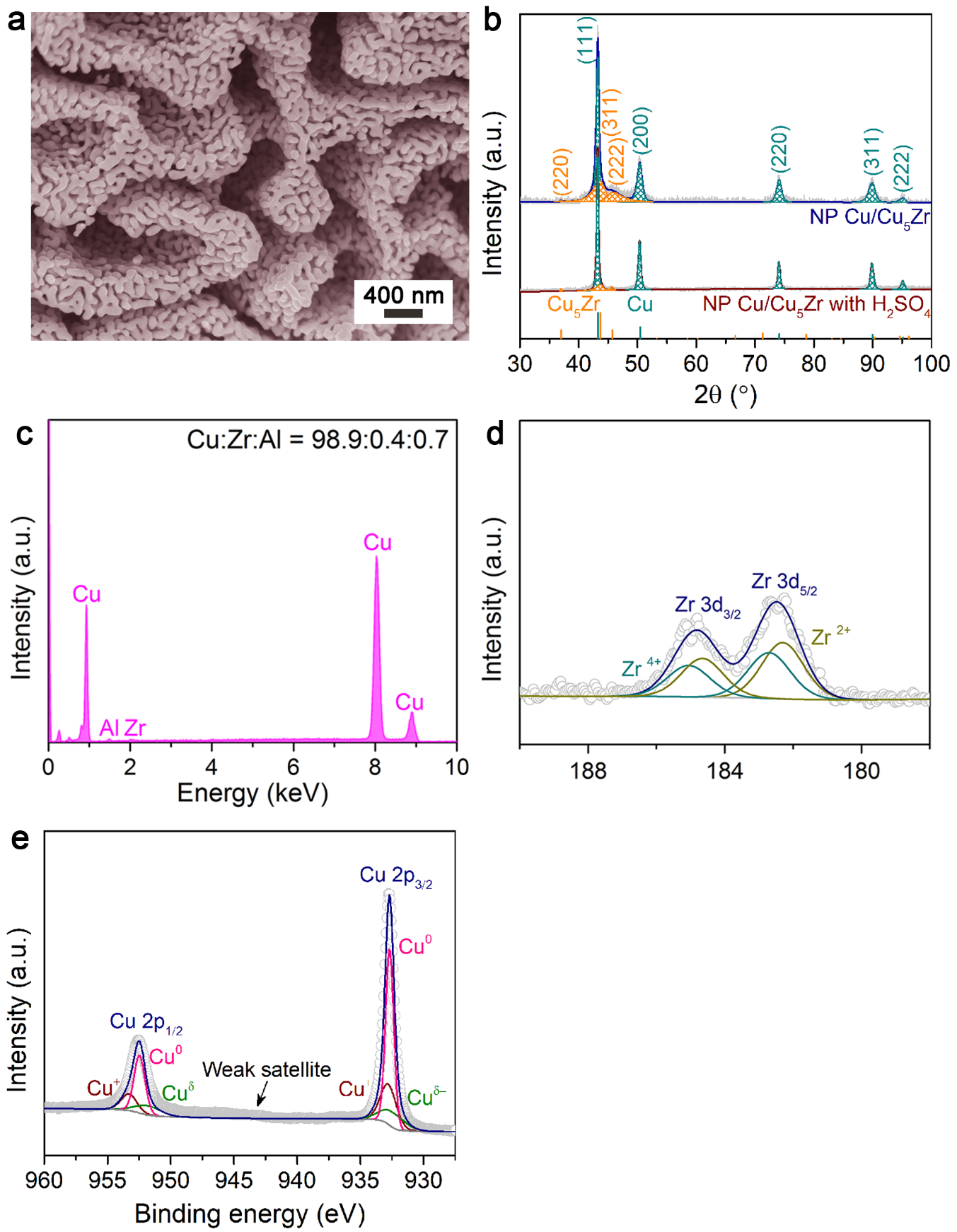


**Figure S15**. **a**, Typical SEM image of NP Cu/Cu_5_Zr electrode after H_2_SO_4_ treatment. **b**, Comparison of XRD patterns of NP Cu/Cu_5_Zr before and after H_2_SO_4_ treatment. **c**, EDS spectrum of NP Cu/Cu_5_Zr electrode after H_2_SO_4_ treatment. **d**, **e**, Zr 3d (d) and Cu 2p (e) XPS spectra of NP Cu/Cu_5_Zr after H_2_SO_4_ treatment.


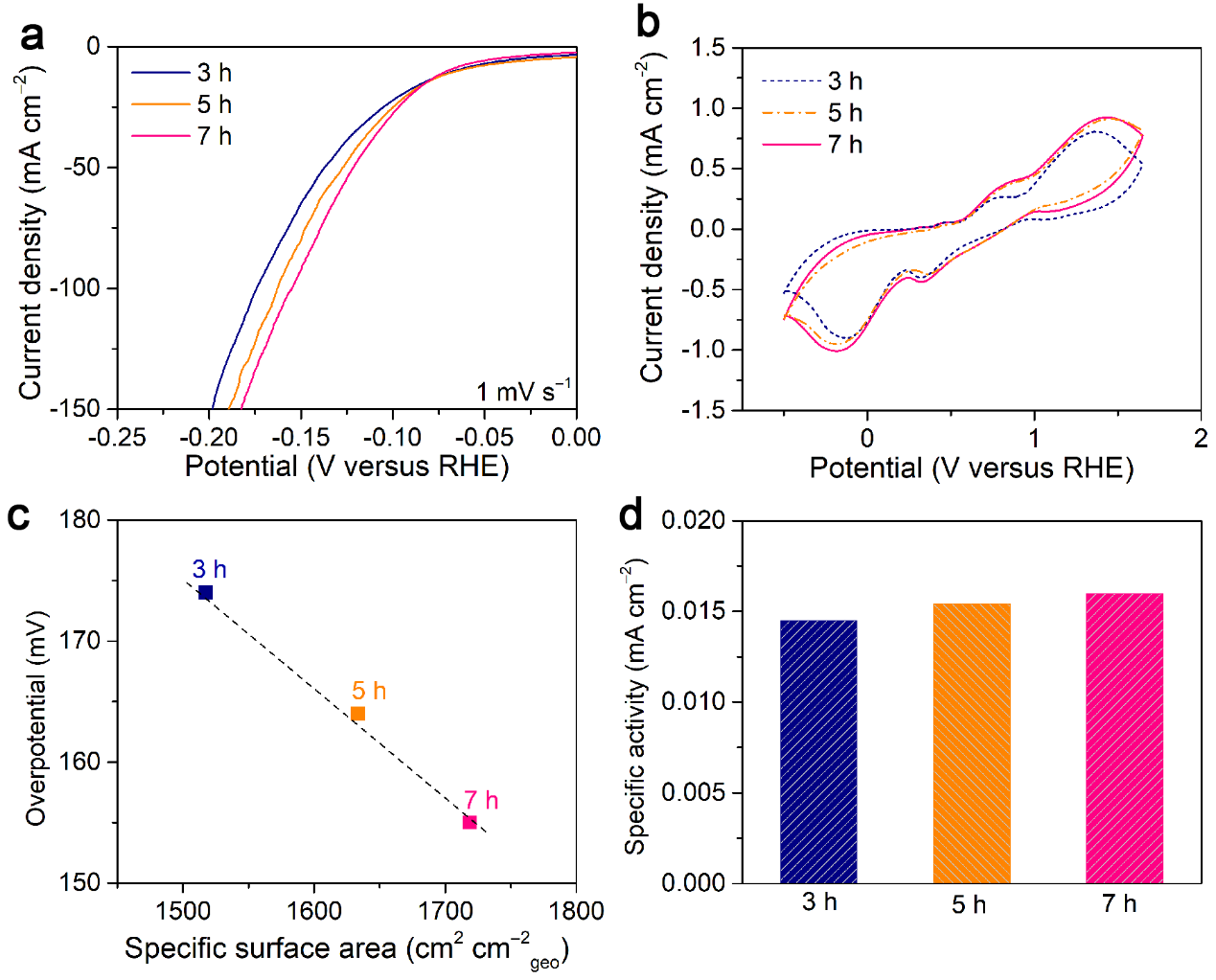


**Figure S16.** **a**, **b**, Typical HER polarization curves (a) and CV curves (b) of NP Cu/Cu_5_Zr electrodes that are fabricated by chemical dealloying precursor Cu_17_Zr_3_Al_80_ alloy for 3 h, 5 h and 7 h at room temperature in 6 M KOH solution, respectively. Therein, the Al component changes from 8.7% to 3.4%. **c**, Overpotential at 100 mA cm^−2^ for the NP Cu/Cu_5_Zr electrodes as a function of specific surface area. **d**, Electrochemical surface area-normalized specific activity of the NP Cu/Cu_5_Zr electrodes that are prepared by chemical dealloying for 3, 5 and 7 h, respectively.

**
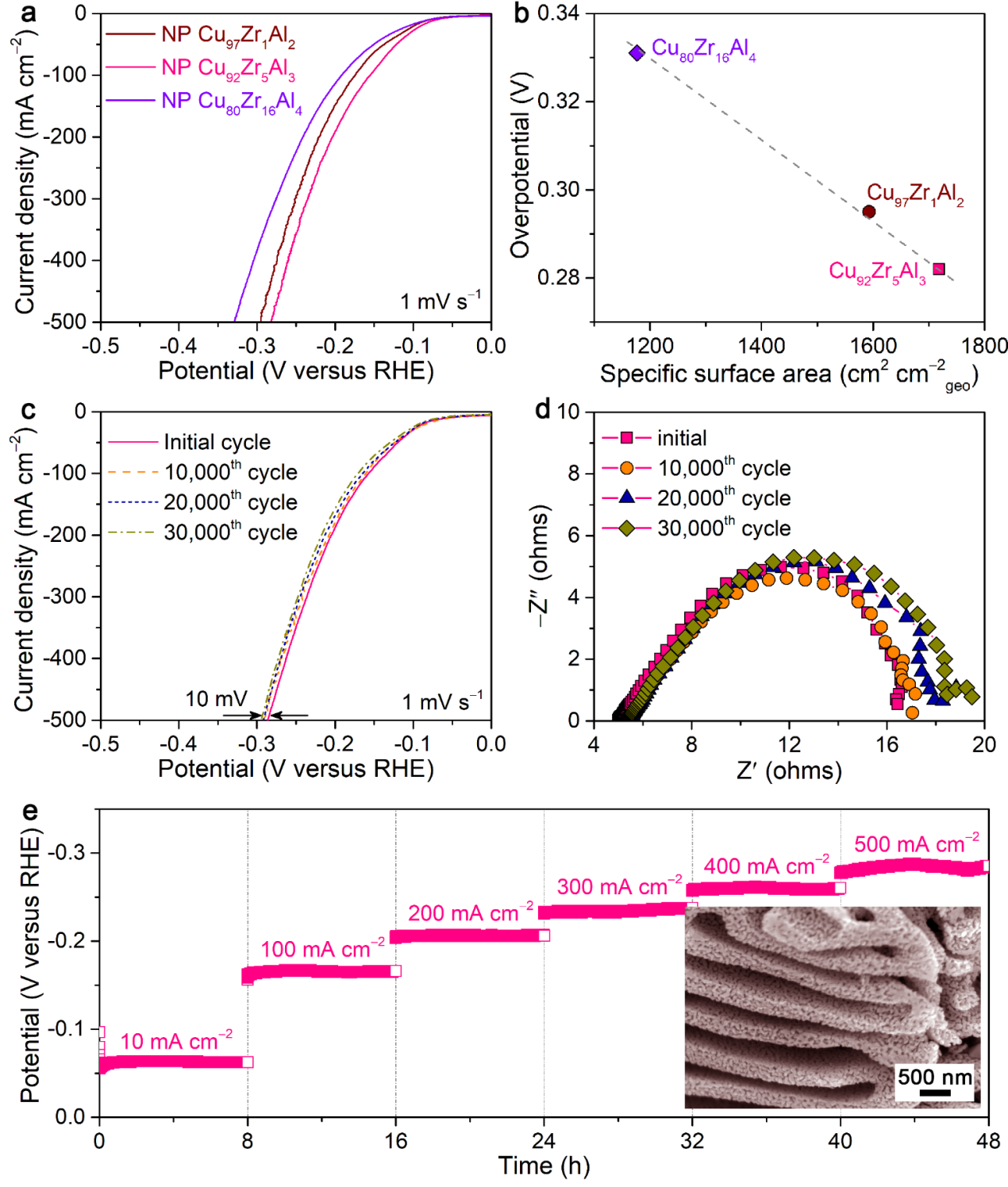
**

**Figure S17.** **a**, Comparison of polarization curves of NP Cu/Cu_5_Zr electrodes with different Cu/Zr/Al compositions, which are fabricated from precursor Cu_20-_*_x_*Zr*_x_*Al_80_ alloys with *x* = 1, 3 and 5. Scan rate of 1 mV s^−1^. Electrolyte: 1 M KOH. **b**, The overpotential at the current density of 500 mA cm^−2^ for NP Cu/Cu_5_Zr as a function of specific surface area.


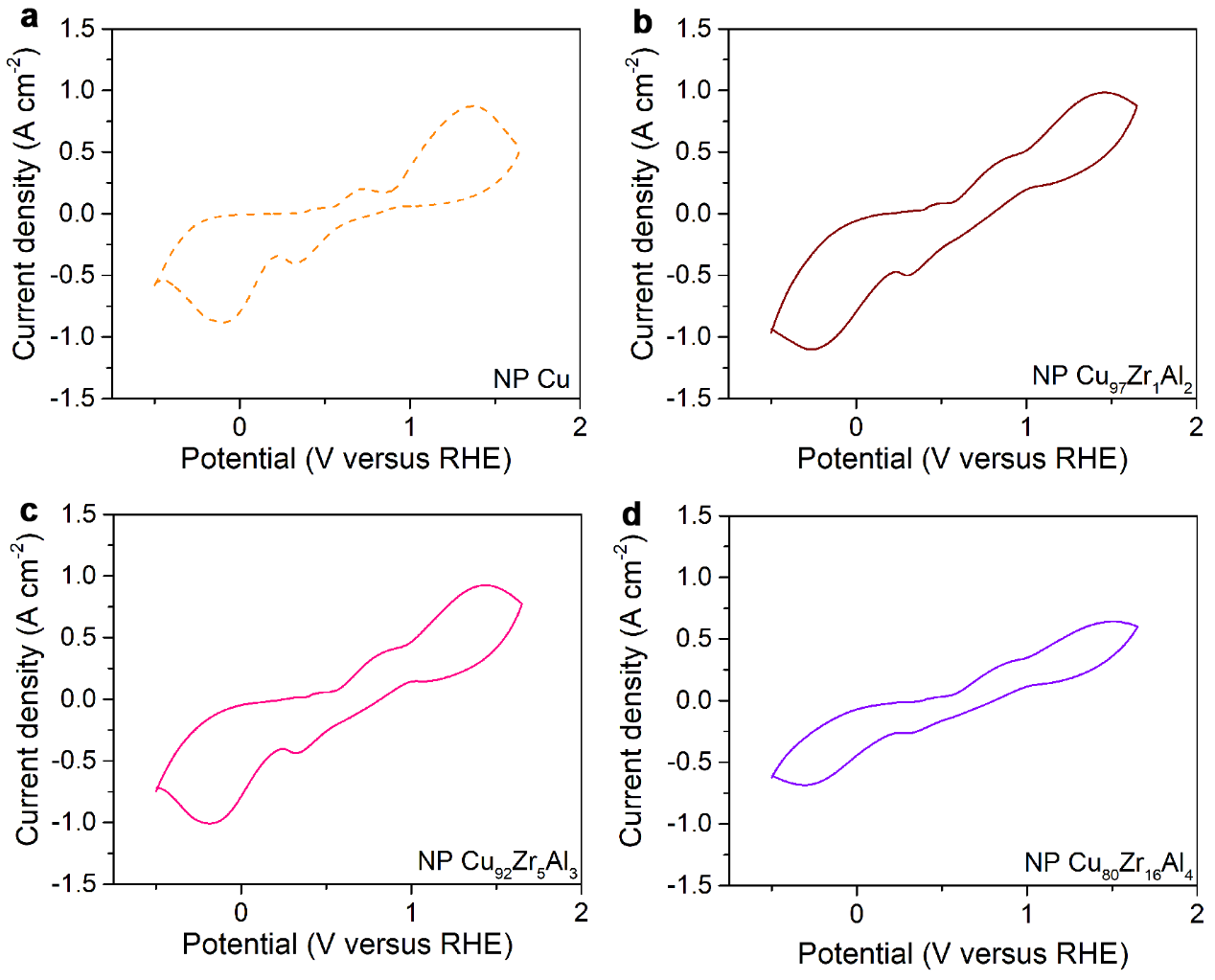


**Figure S18**. Typical CV curves of bare NP Cu (a) and NP Cu/Cu_5_Zr with various Cu/Zr/Al components (b-d) that are fabricated by chemical dealloying Cu_20_Al_80_ and Cu_20-_*_x_*Zr*_x_*Al_80_ (*x* = 1, 3, 5 at%), respectively. Scan rate: 20 mV s^−1^.


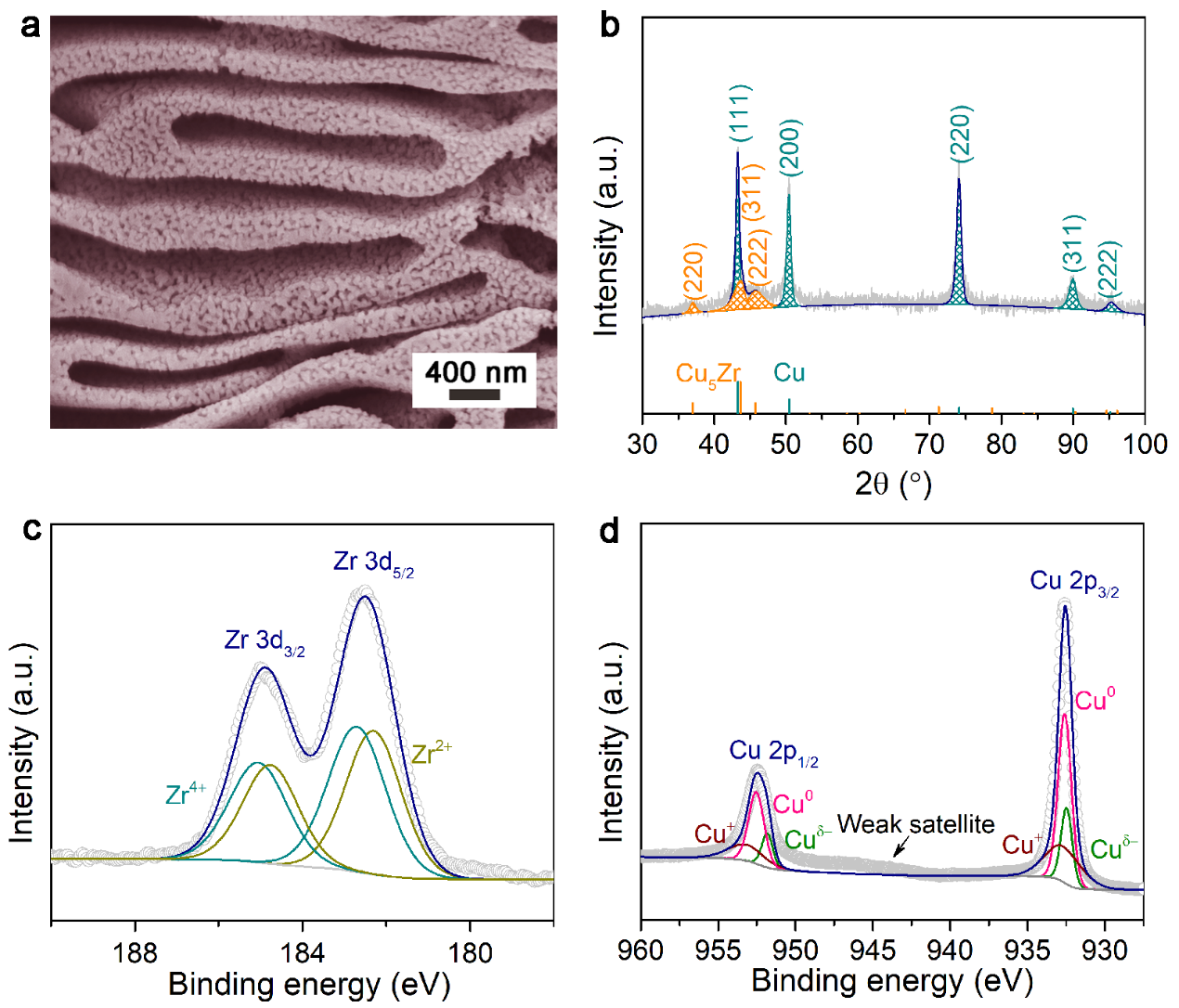


**Figure S19**. **a**, **b**, Typical SEM image (a) and XRD patterns (b) of NP Cu/Cu_5_Zr electrode after potential cycling measurement (10,000 cycles). **c**, **d**, Zr 3d (c) and Cu 2p (d) XPS spectra of NP Cu/Cu_5_Zr after 10,000 cycles.

**Table S1.** The HBE values at the possible adsorption sites on the Cu_5_Zr and Cu_5_Zr-OH illustrated in Figure S2.

| Adsorption sites | Cu_5_Zr | Cu_5_Zr-OH |
| --- | --- | --- |
| No. 1 | −0.44 | −0.36 |
| No. 2 | −0.40 | −0.27 |
| No. 3 | −0.44 | −0.35 |
| No. 4 | −0.44 | −0.36 |
| No. 5 | 0.22 | N/A |

**Table S2.** Chemical compositions of NP Cu/Cu_5_Zr dealloyed from different precursors, which are determined by ICP-MS, EDS and XPS analysis.

| Precursor | NP Cu/Cu_5_Zr | | |
| --- | --- | --- | --- |
|  | ICP-MS  Cu: Zr: Al | EDS  Cu: Zr: Al | XPS  Cu: Zr |
| Cu_19_Zr_1_Al_80_ | 96.9: 1.1: 2.0 | 96.9: 0.9: 2.2 |  |
| Cu_17_Zr_3_Al_80_ | 91.7: 5.0: 3.3 | 92.2: 4.7: 3.1 | 91.9: 5.1 |
| Cu_15_Zr_5_Al_80_ | 80.1: 16.1: 3.8 | 79.9: 16.3: 3.8 |  |

**Table S3.** Comparisons of the catalytic activity of NP Cu/Cu_5_Zr electrocatalytic materials in alkaline medium (pH = 14) with representative noble metal-free ones reported previously.

| Electrocatalysts | *η_ϳ_*  (mA) | *ϳ*  (mA cm^-2^) | Tafel slope  (mV/dec) | Refs. |
| --- | --- | --- | --- | --- |
| NP Cu/Cu_5_Zr | 70  155  203  235  260  280 | 10  100  200  300  400  500 | 68 | This work |
| NP Cu | 150  385 | 10  100 | 172 | This work |
| NP Cu/Cu_5_Zr-O | 108  251 | 10  100 | 168 | This work |
| NP Cu/ZrO_2_ | 119  406 | 10  100 | 152 | This work |
| NiCu@C | 94  247 | 10  100 | 94.5 | 1 |
| Cu/Ni_3_S_2_ | 128  268 | 10  100 | 76.2 | 2 |
| Cu@NiFe LDH | 116  192 | 10  100 | 58.9 | 3 |
| CuCo_2_N_x_@NC | 105  254 | 10  100 | 76 | 4 |
| Cu_3_N-CuO | 118  280  375 | 10  100  300 | 122 | 5 |
| Cu_3_P | 120  255 | 10  100 | 83 | 6 |
| CeO_2_-Cu_3_P | 80  278 | 10  100 | 132 | 7 |
| (Co_0.52_Fe_0.48_)_2_P | 79  180 | 10  100 | 40 | 8 |
| CoMoP@C | 81  160 | 10  100 | 55.53 | 9 |
| P-Co_3_O_4_ | 120  160 | 10  100 | 52 | 10 |
| Ni-Co-P | 107  180  208 | 10  100  200 | 46 | 11 |
| Co_1_Mn_1_CH | 200  340  450 | 10  100  500 | - | 12 |
| NiFeSe@NiSe\|O | 62  225  340 | 10  100  200 | 48.9 | 13 |
| CoP | 87  280  480 | 10  100  200 | 73 | 14 |
| Co-Se | -  268  295 | 10  100  500 | 61.4 | 15 |
| Co-B/Ni | 70  260  410 | 10  100  300 | 68 | 16 |
| MoS_2_/NiCo-LDH | 78  170  200 | 10  100  200 | 76.6 | 17 |
| LaBaSrCoFeO | -  256 | 10  100 | 44 | 18 |
| Fe@FeO_x_S_y_/FeS | -  250  300 | 10  100  200 | 77 | 19 |
| NiCo_2_O_4_ | 110  245  335 | 10  100  200 | 49.7 | 20 |
| TiO_2_/Co | 100  231  281 | 10  100  200 | 62 | 21 |
| CoSe_2_ | 79  185 | 10  100 | 84 | 22 |
| CoSn_2_ | 103  215  365 | 10  100  200 | - | 23 |
| NiCoS_4_ | 210  348 | 10  100 | 58.9 | 24 |
| NPC@MoP | 74  200  300 | 10  100  200 | 52 | 25 |
| Co/NBC | 117  280 | 10  100 | 146 | 26 |
| CoP/NiCoP | 75  270 | 10  100 | 64 | 27 |
| N-NiMoO_4_/NiS_2_ | 99  275 | 10  100 | 74.2 | 28 |
| Ni_0.89_Co_0.11_Se_2_ | 85  160 | 10  100 | 52 | 29 |
| Co@N-C | 108  200 | 10  100 | 55 | 30 |
| Mo_2_N-Mo_2_C | 153  353  530 | 10  100  200 | 68 | 31 |
| δ-FeOOH | 108  180 | 10  100 | 68 | 32 |
| FeCo_/_NiSe_2_ | 92  205  265 | 10  100  200 | 89 | 33 |
| S-CoO | 73  230 | 10  100 | 82 | 34 |
| NiCo/MoS_2_ | 70  230 | 10  100 | 38.1 | 35 |
| *o*-CoSe_2_\|P | 104  180  210 | 10  100  300 | 69 | 36 |
| FeF_2_-F_2_O_3_ | 60  200 | 10  100 | 31 | 37 |
| FeCoNi | 58  215 | 10  100 | 37.5 | 38 |
| Cu-Cu_2_O@C (0.4 M H_2_SO_4_) | 672  990 | 10  100 | 93 | 39 |
| Cu- Cu_2_O (0.5 M KOH) | 210  510 | 10  100 | - | 40 |
| Cu@Cu_x_O (0.1 M KOH) | 200  710 | 10  100 | 61 | 41 |
| Cu_2_Se (0.5 M H_2_SO_4_) | 212  337  544 | 10  100  500 | 33 | 42 |

**Supplementary references**

1. Y. Shen, Y. F. Zhou, D, Wang et al., “Nickel-copper alloy encapsulated in graphitic carbon shells as electrocatalysts for hydrogen evolution reaction,” *Advanced Energy Materials*, vol. 8, no. 2, Article ID 1701759, 2018.
2. J. X. Feng, J. Q. Wu, Y. X. Tong et al., “Efficient hydrogen evolution on Cu nanodots-decorated Ni_3_S_2_ nanotubes by optimizing atomic hydrogen adsorption and desorption,” *Journal of the Americal Chemical Society*, vol. 140, no. 2, pp. 610-617, 2018.
3. L. Yu, H. Q. Zhou, J. Y. Sun et al., “Cu nanowires shelled with NiFe layered double hydroxide nanosheets as bifunctional electrocatalysts for overall water splitting,” *Energy & Environmental Science*, vol. 10, no. 8, pp. 1820-1827, 2017.
4. J. Zheng, X. L. Chen, X. Zhong et al., “Hierachical porous NC@CuCo nitride nanosheet networks: Highly efficient bifunctional electrocatalyst for overall water splitting and selective electrooxidation of benzyl alcohol,”*Advanced Functional Materials*, vol. 27, no. 46, Article ID 1704169, 2017.
5. C. Panda, P. W. Menezes, M. Zheng et al., “In situ formation of nanostructured core-shell Cu_3_N-CuO to promote alkaline water electrolysis,” *ACS Energy Letters,* vol. 4, no. 3, pp. 747-754, 2019.
6. J. H. Hao, W. S. Yang, Z. P. Huang et al., “Superhydrophilic and superaerophobic copper phosphide microsheets for efficient electrocatalytic hydrogen and oxygen evolution,” *Advanced Materials Interfaces*, vol. 3, no. 16, Article ID 1600236, 2016.
7. Z. Wang, H. T. Du, Z. A. Liu et al., “Interface engineering of a CeO_2_-Cu_3_P nanoarray for efficient alkaline hydrogen evotion,” *Nanoscale,* vol. 10, no. 5, pp. 2213-2217, 2018
8. Y. W. Tan, H. Wang, P. Liu et al., “Versatile nanoporous bimetallic phosphide towards electrochemical water splitting,” *Energy & Environmental Science*, vol. 9, no. 7, pp. 2257-2261, 2016.
9. Y. Y. Ma, C. X. Wu, X. J. Feng et al., “Highly efficient hydrogen evolution from seawater by a low-cost and stable CoMoP@C electrocatalyst superior to Pt/C,” *Energy & Environmental Science*, vol. 10, no. 3, pp. 788-798, 2017.
10. Z. H. Xiao, Y. Wang, Y. C. Huang et al., “Filling the oxygen vacancies in Co_3_O_4_ with phosphorus: an ultra-efficient electrocatalyst for overall water splitting,” *Energy & Environmental Science*, vol. 10, no. 12, pp. 2563-2569, 2017.
11. E. L. Hu, Y. F. Feng, J. W. Nai et al., “Construction of hierarchical Ni–Co–P hollow nanobricks with oriented nanosheets for efficient overall water splitting,” *Energy & Environmental Science*, vol. 11, no. 4, pp. 872-880, 2018.
12. T. Tang, W. J. Jiang, S. Niu et al., “Electronic and morphological dual modulation of cobalt carbonate hydroxides by Mn doping toward highly efficient and stable bifunctional electrocatalysts for overall water splitting,” *Journal of the Americal Chemical Society*, vol. 139, no. 24, pp. 8320-8328, 2017.
13. G. Yilmaz, C. F. Tan, Y. F. Lim et al., “Pseudomorphic transformation of interpenetrated Prussian blue analogs into defective nickel iron selenides for enhanced electrochemical and photo-electrochemical water splitting,” *Adv. Energy Mater*ials, vol. 9, no. 1, Article ID 1802983, 2019.
14. X. T. Yu, M. Y. Wang, X. Z. Gong et al., “Self-supporting porous CoP-based films with phase-separation structure for ultrastable overall water electrolysis at large current density,” *Adv. Energy Mater*ials, vol. 8, no. 34, Article ID 1802445, 2018.
15. Y. Q. Zhao, B. Jin, Y. Zheng et al., “Charge state manipulation of cobalt selenide catalyst for overall seawater electrolysis,” *Adv. Energy Mater*ials, vol. 8, no. 29, Article ID 1801926, 2018.
16. W. J. Hao, R. B. Wu, R. Q. Zhang et al., “Electroless plating of highly efficient bifunctional boride based electrodes toward practical overall water Splitting,” *Adv. Energy Mater*ials, vol. 8, no. 26, Article ID 1801372, 2018.
17. J. Hu, C. X. Zhang, L. Jiang et al., “Nanohybridization of MoS_2_ with layered double hydroxides efficiently synergizes the hydrogen evolution in alkaline media,” *Joule*, vol. 1, no. 2, pp. 383-393, 2017.
18. B. Hua, M. Li, W. Y. Pang et al., “Activating *p*-blocking centers in perovskite for efficient water splitting,” *Chem*, vol. 4, no. 12, pp. 2902-2916, 2018.
19. X. X. Zou, Y. Y. Wu, Y. P. Liu et al., “In situ generation of bifunctional, efficient Fe-based catalysts from mackinawite iron sulfide for water splitting,” *Chem*, vol. 4, no. 5, pp. 2902-2916, 2018.
20. X. H. Gao, H. X. Zhang, Q. G. Li et al., “Hierarchical NiCo_2_O_4_ hollow microcuboids as bifunctional electrocatalysts for overall water-splitting,”*Angewante Chemie-International Edition*, vol. 55, no. 21, pp. 6290-6294, 2016.
21. J. X. Feng, H. Xu, Y. T. Dong et al., “Efficient hydrogen evolution electrocatalysis using cobalt nanotubes decorated with titanium dioxide nanodots,”*Angewante Chemie-International Edition*, vol. 56, no. 11, pp. 2960-2964, 2017.
22. J. Y. Zhang, H. M. Wang, Y. F. Tian et al., “Anodic hydrazine oxidation assists energy-efficient hydrogen evolution over a bifunctional cobalt perselenide nanosheet electrode,”*Angewante Chemie-International Edition*, vol. 57, no. 26, pp. 7649-7653, 2018.
23. P. W. Menezes, C. Panda, S. Garai et al., “Structurally ordered intermetallic cobalt stannide nanocrystals for high-performance electrocatalytic overall water-splitting,”*Angewante Chemie-International Edition*, vol. 57, no. 46, pp. 15237-15242, 2018.
24. A. Sivanantham, P. Ganesan, and S. Shanmugam et al., “Hierarchical NiCo_2_S_4_ nanowire arrays supported on Ni foam: An efficient and durable bifunctional electrocatalyst for oxygen and hydrogen evolution reactions,” *Advanced Functional Mater*ials, vol. 26, no. 26, pp. 4661-4672, 2016.
25. B. C. Liu, H. Li, B. Cao et al., “Few layered N, P dual-doped carbon-encapsulated ultrafne MoP nanocrystal/MoP cluster hybrids on carbon cloth: An ultrahigh active and durable 3D self-supported integrated electrode for hydrogen evolution reaction in a wide pH range,” *Advanced Functional Mater*ials, vol. 28, no. 30, Article ID 1801527, 2018.
26. M. R. Liu, Q. L. Hong, Q. H. Li et al., “Cobalt boron imidazolate framework derived cobalt nanoparticles encapsulated in B/N codoped nanocarbon as effcient bifunctional electrocatalysts for overall water splitting,” *Advanced Functional Mater*ials, vol. 28, no. 26, Article ID 1801136, 2018.
27. R. Boppella, J. W. Tan, W. Yang et al., “Homologous CoP/NiCoP heterostructure on N-Doped carbon for highly efficient and pH-universal hydrogen evolution electrocatalysis,” *Advanced Functional Mater*ials, vol. 29, no. 6, Article ID 1807976, 2019.
28. L. An, J. R. Feng, Y. Zhang et al., “Epitaxial heterogeneous interfaces on N-NiMoO_4_/NiS_2_ nanowires/nanosheets to boost hydrogen and oxygen production for overall water splitting,” *Advanced Functional Mater*ials, vol. 29, no. 1, Article ID 1805298, 2019
29. B. Liu, Y. F. Zhao, H.Q. Peng et al., “Nickel–cobalt diselenide 3D mesoporous nanosheet networks supported on Ni foam: An all-pH highly effcient integrated electrocatalyst for hydrogen evolution,” *Advanced Mater*ials, vol. 29, no. 19, Article ID 1606521, 2017.
30. Z. L. Chen, R. B. Wu, Y. Liu et al., “Ultrafne Co nanoparticles encapsulated in carbon nanotubes-grafted graphene sheets as advanced electrocatalysts for the hydrogen evolution reaction,” *Advanced Mater*ials, vol. 30, no. 30, Article ID 1802011, 2018.
31. H. J. Yan, Y. Xie, Y. Q. Jiao et al., “Holey Reduced graphene oxide coupled with an Mo_2_N–Mo_2_C heterojunction for efficient hydrogen evolution,” *Advanced Mater*ials, vol. 30, no. 2, Article ID 1704156, 2018.
32. B. Liu, Y. Wang, H. Q. Peng et al., “Iron vacancies induced bifunctionality in ultrathin feroxyhyte nanosheets for overall water splitting,” *Advanced Mater*ials, vol. 30, no. 36, Article ID 1803144, 2018.
33. Y. Q. Sun, K. Xu, Z. X. Wei et al., “Strong electronic interaction in dual-cation-incorporated NiSe_2_ nanosheets with lattice distortion for highly efficient overall water splitting,” *Advanced Mater*ials, vol. 30, no. 35, Article ID 1802121, 2018.
34. T. Ling, D. Y. Yan, H. Wang et al., “Activating cobalt(II) oxide nanorods for efficient electrocatalysis by strain engineering,” *Nature Commun*ications, vol. 8, Article ID 1509, 2017.
35. H. Y. Li, S. M. Chen, X. F. Jia et al., “Amorphous nickel-cobalt complexes hybridized with 1T-phase molybdenum disulfide via hydrazine induced phase transformation for water splitting,” *Nature Commun*ications, vol. 8, Article ID 15377, 2017.
36. Y. R. Zheng, P. Wu, M. R. Gao et al., “Doping-induced structural phase transition in cobalt diselenide enables enhanced hydrogen evolution catalysis,” *Nature Commun*ications, vol. 9, Article ID 2533, 2018.
37. X. J. Fan, Y. Y. Liu, S. Chen et al., “Defect-enriched iron fluoride-oxide nanoporous thin films bifunctional catalyst for water splitting,” *Nature Commun*ications, vol. 9, Article ID 1809, 2018.
38. H. Y. Li, S. M. Chen, Y. Zhang et al., “Systematic design of superaerophobic nanotube array electrode comprised of transition-metal sulfides for overall water splitting,” *Nature Commun*ications, vol. 9, Article ID 2452, 2018.
39. P. Muthukumar, V. V.Kumar, G. R. K. Reddy et al., “Fabrication of strong bifunctional electrocatalytically active hybrid Cu-Cu_2_O nanoparticles in a carbon matrix,” *Catalysis Science & Technol*ogy, vol. 8, no. 5, pp. 1414-1422, 2018.
40. B. Kumar, S. Saha, K. Ojha et al., “A facile one step synthesis of Cu/Cu_2_O nanocomposites: Enhanced hydrogen/oxygen evolution,” *Materials Research Bulletin*, vol. 64, pp. 283-287, 2015.
41. D. T. Tran, H. T. Le, T. L. L.Doan et al., “Pt nanodots monolayer modified mesoporous Cu@Cu_x_O nanowires for improved overall water splitting reactivity,” *Nano Energy*, vol. 59, pp. 216-228, 2019.
42. S. Anantharaj, T. S. Amarnath, E. Subhashini et al., “Shrinking the hydrogen overpotential of Cu by 1 V and imparting ultralow charge transfer resistance for enhanced H_2_ evoliton,” *ACS Catal*ysis , vol. 8, no. 7, pp. 5686-5697, 2018.
